# CROPGRIDS: a global geo-referenced dataset of 173 crops

**DOI:** 10.1038/s41597-024-03247-7

**Published:** 2024-04-22

**Authors:** Fiona H. M. Tang, Thu Ha Nguyen, Giulia Conchedda, Leon Casse, Francesco N. Tubiello, Federico Maggi

**Affiliations:** 1 School of Environmental and Rural Science, University of New England, Armidale, New South Wales 2351 Australia; 2 Department of Civil Engineering, Monash University, Clayton, 3800 Victoria Australia; 3 Environmental Engineering, School of Civil Engineering, The University of Sydney, Sydney, New South Wales Australia; 4 Statistics Division, Food and Agriculture Organization of the United Nations, Viale delle Terme di Caracalla, Rome, 00153 Italy; 5 Sydney Institute of Agriculture, The University of Sydney, Sydney, NSW 2006 Australia

**Keywords:** Agriculture, Environmental sciences

## Abstract

CROPGRIDS is a comprehensive global geo-referenced dataset providing area information for 173 crops for the year 2020, at a resolution of 0.05° (about 5.6 km at the equator). It represents a major update of the Monfreda *et al*. (2008) dataset (hereafter MRF), the most widely used geospatial dataset previously available, covering 175 crops with reference year 2000 at 10 km spatial resolution. CROPGRIDS builds on information originally provided in MRF and expands it using 27 selected published gridded datasets, subnational data of 52 countries obtained from National Statistical Offices, and the 2020 national-level statistics from FAOSTAT, providing more recent harvested and crop (physical) areas for 173 crops at regional, national, and global levels. The CROPGRIDS data advance the current state of knowledge on the spatial distribution of crops, providing useful inputs for modelling studies and sustainability analyses relevant to national and international processes.

## Background & Summary

Detailed global geospatial information on the distribution of crop types over time is required to understand planetary boundaries and support decision-making at all scales, from land use change dynamics to the impacts of agricultural inputs on the environment. Geo-referenced crop information is particularly valuable for improving reporting and monitoring progress at sub-national scales under the Sustainable Development Goals (SDG), in particular Goal 2 indicators on the productivity and sustainability of agriculture^1^.

The most comprehensive geospatial product available today, covering 175 crops at a resolution of about 10 km globally^2^—henceforth referred to herein as MRF from the initials of the authors—provides however dated information, limited to the year 2000, whereas significant changes in cropland extent have been documented over the past twenty years^3,4^. MRF was created by spatially disaggregating official national and sub-national harvested area information obtained from various sources, over a gridded cropland map derived from remote sensing. It has since been used in several published studies, most notably for assessing planetary boundaries with respect to food and agriculture^5^. Several crop type mapping efforts were made since the production of MRF (see ref. ^6^ for a comprehensive review). More recently, important initiatives such as those promoted by the European Space Agency (ESA)^7,8^, by the USA National Aeronautics and Space Administration (NASA)^9^, and by the G20 Ministers of Agriculture were launched and are already contributing considerable new information^10–13^. However, none of these efforts has matched the original MRF scope and crop coverage, so much so that many global assessments of agricultural impacts have continued to use MRF as a reference^14–17^.

To update the MRF information, we produced CROPGRIDS, a new global gridded harvested and crop (physical) area geospatial dataset for 173 crops for the year 2020. CROPGRIDS was produced using a similar approach as in ref. ^13^ with MRF data used as starting point and updated through hybridisation of more recent information, by merging all available, published and gridded datasets for periods more recent than 2000 and using a set of endogenous and exogenous data quality indicators, within a multi-criteria ranking scheme, to determine best-fit data by crop type and country. For some crop types and countries where gridded data more recent than 2000 were not available, we spatialized recent subnational data obtained from National Statistical Offices (NSOs) following a similar algorithm used in MRF but with a new cropland agreement map circa 2020^18^ as the cropland mask. The resulting CROPGRIDS is a novel synthesis of the most recently available information on harvested and crop area maps for 173 crops, at a global spatial resolution of 0.05° (approximately 5.6 km at the equator). Crop type name, harvested area and crop area definitions used in CROPGRIDS are aligned to the relevant FAO commodities and land use definitions^19^.

## Methods

**Fig. 1:**
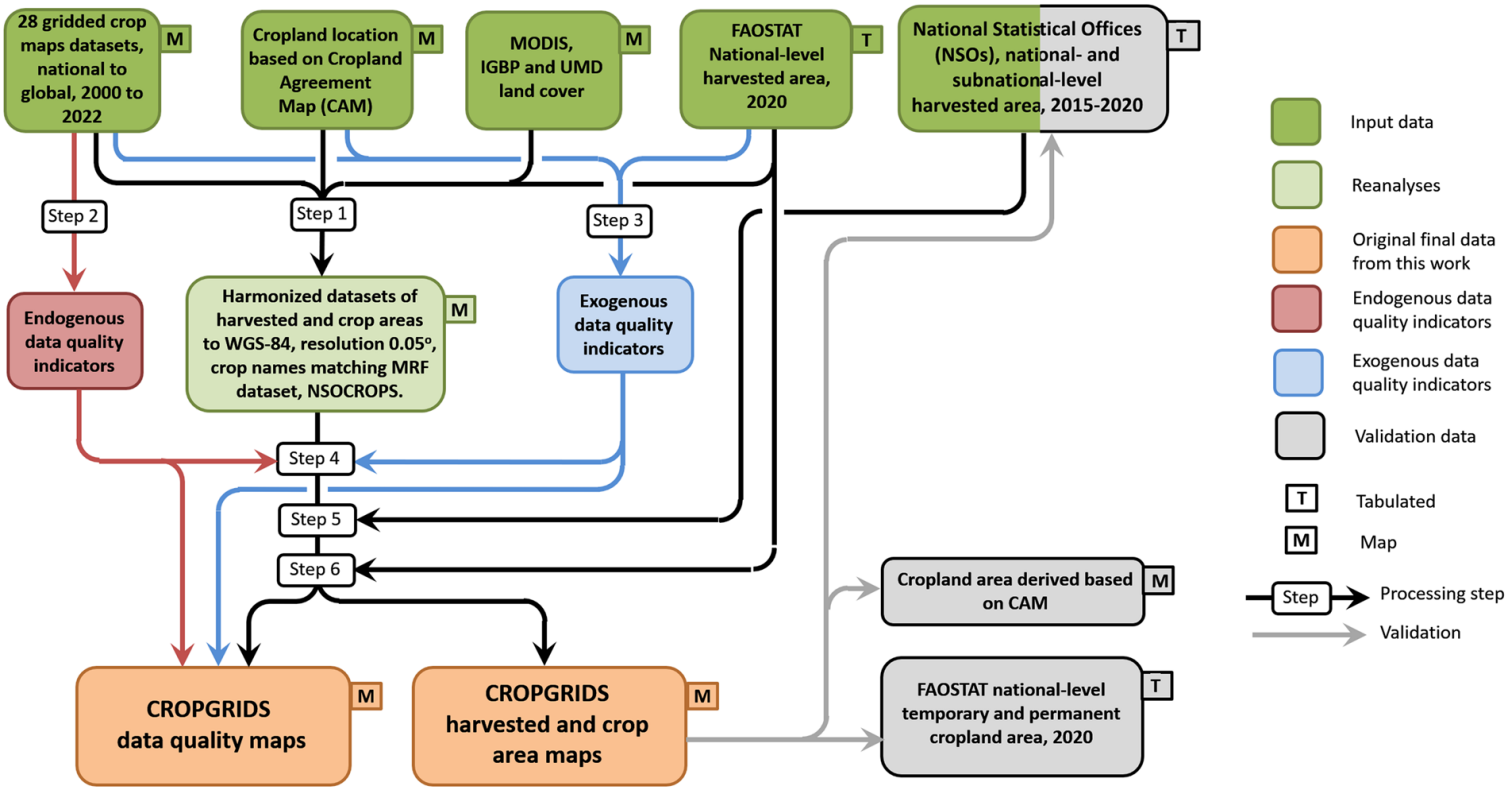
Workflow of the development of CROPGRIDS. Step 1: Input data harmonization; Step 2: computation of endogenous data quality indicators; Step 3: computation of exogenous data quality indicators; Step 4: assemblage of global maps; Step 5: gap filling of crop geographic distribution; and Step 6: data adjustment.

The development of CROPGRIDS involves several steps that were carried out either sequentially or in parallel (Fig. 1) as follows: Step 1) input data harmonization; Step 2) computation of endogenous data quality indicators; Step 3) computation of exogenous data quality indicators; Step 4) assemblage of global maps; Step 5) gap filling of crop geographic distribution; and Step 6) data adjustment to FAOSTAT. These steps are described in detail in the next sections. While Step 1) to 3) are mostly data curation and pre-calculations for later steps, Step 4), at the core of the workflow, was achieved through a multi-criteria ranking scheme designed using the endogenous and exogenous data quality indicators to select, for countries and territories for which data were available from multiple input datasets, the one dataset best describing a specific crop. Similarly important, Steps 5) and 6) were next used to update missing information using existing independent subnational statistics and adjust the assembled data maps to the FAOSTAT reference year 2020.

### Input data

We conducted a search for published peer-reviewed datasets providing geo-referenced crop-specific information, including, by grid cell: amount of harvested area (*HA*); amount of crop area (*CA*); fractional crop area (*f*, *i.e*., proportion of the grid cell area occupied by crop type); or binary values (*w*), specifying whether a grid cell was cultivated with a specific crop type or not. The following four criteria were applied for inclusion of a dataset: (1) reference year later than 2000; (2) at least one crop species also present in MRF; (3) geospatial coverage for at least one country (complete national extent); and (4) spatial resolution at least 0.083° (about 10 km at the equator). Based on these criteria, we created a library of 28 datasets, including 14 national, 8 multinational/continental, and 6 global datasets (Table 1). Amongst the selected datasets, two provided both *HA* and *CA*, two provided *HA*, three provided *f*, and 21 provided *w* (see details in Table 1). The information collected to build CROPGRIDS spanned the period 2000–2021, with 25 out of the 28 input datasets referring to the period 2015–2021. These datasets provided diverse variables and had different resolution from the target resolution of 0.05° per grid cell for CROPGRIDS. Hence, a number of steps were undertaken to harmonize the input datasets as described in the section below.

**Table 1:** CROPGRIDS input datasets.

	Acronym	Description	Reference
1	MRF	Global gridded *HA* [ha] for 175 crops at a resolution of 0.0833 degree (~10 km at the equator) in 2000.	^2^
2	SPAM	Global gridded *HA* and *CA* [ha] for 38 crops at a resolution of 0.0833 degree (~10 km at the equator) in 2010. Only 30 crops considered for this work.	^29^
3	GAEZ + 2015	Global gridded *HA* [ha] for 26 crops at a resolution of 0.0833 degree (~10 km at the equator) in 2015. Only 20 crops considered.	^30^
4	GEOGLAM	Global gridded *f* [%] for 4 crops at a resolution of 0.05 degree (~5.6 km at the equator) in 2020.	^13,31^
5	OIPA	Global gridded *w* [−] for oil palm at a resolution of 0.0000898 degree (~0.01 km at the equator) in 2019.	^32^
6	RAP	Global gridded *w* [−] for rapeseed at a resolution of 0.0000898 degree (~0.01 km at the equator) in 2019.	^33^
7	EU	Gridded *w* [−] for 28 countries in EU for 17 crops at a resolution of 0.0000898 degree (~0.01 km at the equator) in 2018. Only 12 crops considered.	^34^
8	SPAMAF	Gridded *HA* and *CA* [ha] for Africa for 42 crops at a resolution of 0.0833 degree (~10 km at the equator) in 2017. Only 34 crops considered.	^35^
9	AFCAS	Gridded *f* [ha km^−2^] for Africa for cassava at a resolution of 0.00833 degree (~1 km at the equator) in 2014.	^36^
10	SASOY	Gridded *w* [−] for South America for soybean at a resolution of 0.00025 degree (~0.03 km at the equator) in 2018.	^37^
11	MYSTHA	Gridded *w* [−] for Malaysia, Indonesia, and Thailand for oil palm at a resolution of 0.0002695 degree (~0.03 km at the equator) in 2017.	^38^
12	ASIARICE	Gridded *w* [−] and cropping intensity for 21 countries in Asian monsoon region for rice at a resolution of 0.0045 degree (~0.5 km at the equator) in 2020.	^39^
13	CIVGHA	Gridded *w* [−] for Cote d’Ivoire and Ghana for cocoa at a resolution of 0.0000898 degree (~0.01 km at the equator) in 2019.	^40^
14	UZBTJK	Gridded *w* [−] for Uzbekistan and Tajikistan for 38 crops distributed as shapefile at a resolution of 0.0001 degree (~0.01 km at the equator) in 2015 to 2018. Only 20 crops considered.	^41^
15	USA	Gridded *w* [−] for USA for 105 crops at a resolution of 0.0000898 degree (~0.01 km at the equator) in 2021. Only 64 crops considered.	^42^
16	CA	Gridded *w* [−] for Canada for 52 crops at a resolution of 0.00027 degree (~0.03 km at the equator) in 2021. Only 31 crops considered.	^43^
17	AFG	Gridded *w* [−] for Afghanistan for 6 crops at a resolution of 0.0000898 degree (~0.01 km at the equator) in 2020. Only 3 crops considered.	^44^
18	DEU	Gridded *w* [−] for Germany for 24 crops at a resolution of 0.0000898 degree (~0.01 km at the equator) in 2019. Only 15 crops considered.	^45^
19	CHNWH	Gridded *w* [−] for China for winter wheat at a resolution of 0.0003 degree (~0.03 km at the equator) for 2018.	^46^
20	CHNMZ	Gridded *w* [−] for China for maize at a resolution of 0.005 degree (~0.56 km at the equator) in 2017.	^47^
21	CHNMZWHRI	Gridded *w* [−] of single, double, triple cropping for China for rice, maize, and wheat at a resolution of 0.005 degree (~0.56 km at the equator) in 2020.	^48^
22	BGDRICE	Gridded *w* [−] of 3 growing seasons for Bangladesh for rice at a resolution of 0.0000898 degree (~0.01 km at the equator) in 2017.	^49^
23	BRA	Gridded *w* [−] for Brazil for sugarcane at a resolution of 0.0003 degree (~0.03 km at the equator) in 2019.	^50^
24	SEN	Gridded *w* [−] for Senegal for 22 crops at a resolution of 0.00009 degree (~0.01 km at the equator) in 2018. Only 17 crops considered.	^51^
25	AU	Gridded *f* [−] for Australia for 25 crops at a resolution of 0.0833 degree (~10 km at the equator) in 2015. Only 6 crops considered.	^52^
26	FR	Gridded *w* [−] for France for 11 crops at a resolution of 0.0001 degree (~0.01 km at the equator) in 2021. Only 5 crops considered.	^53^
27	JP	Gridded *w* [−] for Japan for rice at a resolution of 0.0000833 degree (~0.01 km at the equator) in 2020.	^54^
28	CHNMZSOY	Gridded *w* [−] for China for maize and soybean at a resolution of 0.0000833 degree (~0.01 km at the equator) in 2019.	^55^

Variables provided in datasets are: *HA*, harvested area; *CA*, crop (physical) area; *f*, fractional crop area; and *w*, binary value for a crop existence in a grid cell. Aggregated crops that cannot be matched against the crop list in MRF were excluded. The full list of crop name matching is provided in Supplementary Table S2.

Additionally, we used the following datasets for data processing: a cropland agreement map (CAM) circa year 2020 at 30 m resolution^18^; the MODIS land use maps for year 2020 at 500 m resolution^20^; the FAO Global Administrative Unit Layers (GAUL) dataset^21^ (FAO, 2015); subnational statistics from various NSOs (see Supplementary Table S1) and FAOSTAT national statistics of harvested area^19^. CAM provides geospatial statistics of cropland areas, generated based on six open-access high-resolution remote sensing products^18^.

### Input data harmonization (Step 1)

We first determined *HA* and *CA* for each crop type by grid cell for each input datasets listed in Table 1 as follows.

When only *HA* data are given (i.e., MRF and GAEZ + 2015, Table 1), we imputed *CA* = *HA* for permanent crops and *CA* = min{*HA*, *LA, CAM95*} for temporary crops (see list of permanent and temporary crops in Supplementary Table S3), with *LA* being the area of land available for cropping, i.e., grid cell area (*GA*) excluding water bodies, wetlands, urban and built-up lands, permanent snow and ice, and barren land following land use classification in MODIS^20^, and *CAM95* being the 95^th^ percentile surface area calculated from CAM. When only *f* was provided (i.e., GEOGLAM, AFCAS, and AU), we calculated crop area as *CA* = *f* × *GA* and imputed *HA* = *CA*. Next, the computed and original georeferenced maps of *HA* and *CA* in MRF, SPAM, GAEZ + 2015, and SPAMAF datasets were harmonized to a common spatial resolution of 0.05° (approximately 5.6 km at the equator) using the *imresize* function^22^ in Matlab with bilinear interpolation and pychnophylactic methods to ensure areal conservation, and a bounding box of −180° to 180° longitude and −90° to 90° latitude using the WGS-84 coordinate system (World Geodetic System 1984).

When only *w* was provided, we first derived corresponding *f* values and then made the same imputations as above. Specifically, since datasets providing *w* for individual crops had typically high spatial resolution (ranging 10–550 m at the equator), we performed pixel counting of *w* values to derive *f* values at the required 5.6 km resolution (i.e., 0.05° per grid cell). Additionally, for datasets providing *w* values of a specific crop over multiple growing seasons *s*, the annual *CA* of that crop was computed as *CA* = max{*CA*_*1*_, *CA*_*2*_, …, *CA*_*s*_} across the seasons *s*; while *HA* was computed as $HA=\mathop{\sum }\limits_{i=1}^{s}{CA}_{i}$. Alternatively, when the geo-referenced cropping intensity *CI* was provided (i.e., ASIARICE, Table 1), then we calculated *HA* = *CA* × *CI*.

Finally, we set a threshold for *CA* and *HA* values, i.e., both were set to zero whenever *CA* < 100 m^2^. This lower bound corresponds to the finest spatial resolutions of all input datasets and was set to prevent from accounting of unrealistically small agriculture parcels. Consistency diagnostics checked that *CA* ≤ *HA*, *CA* ≤ *LA ≤ GA*, and *CI* ≤ 3 (i.e., *CI* commonly less than 3^23^) were satisfied in all grid cells for individual crops.

In building CROPGRIDS, we also harmonized crop names in the input datasets, including performing aggregations where needed, to correspond to the crop names in MRF, thus ensuring internal consistency and alignment with FAO crop classifications following the Indicative Crop Classification (ICC) of the World Programme for the Census of Agriculture^24^ (Supplementary Table S2).

### Compute endogenous data quality indicators (Step 2)

Endogenous data quality indicators assessed both quantitative and qualitative features of a dataset that do not depend on external information. These endogenous features included: synchrony (*Q*_*y*_), administration (*Q*_*a*_), data source (*Q*_*s*_), validation (*Q*_*v*_), resolution (*Q*_*r*_), maturity (*Q*_*m*_), and type of dispatch (*Q*_*d*_). All endogenous features were assigned an indicator value ranging 0–1, with the end points corresponding to the lowest and highest quality, respectively. Endogenous features were not expressed as geo-referenced maps, but rather we used them to tag individual input datasets regardless of crop type.

*Q*_*y*_ described the level of synchrony between the year of reference *Y*_*r*_ of a dataset and the year of reference of CROPGRIDS, which was set to 2020. Specifically, datasets with *Y*_*r*_ departing from 2020 were assigned a lower rank than those in 2020 as

1 $${Q}_{y}=\{\begin{array}{cc}\frac{{Y}_{r}-2000}{2020-2000} & {\rm{i}}{\rm{f}}\,{Y}_{r} < 2020\\ 1 & {\rm{i}}{\rm{f}}\,{Y}_{r}=2020\\ 1-\frac{{Y}_{r}-2020}{2040-2020} & {\rm{i}}{\rm{f}}\,{Y}_{r} > 2020\end{array}$$

*Q*_*a*_ described the administrative domain of a dataset (i.e., national to global). A national dataset was assigned a higher *Q*_*a*_ value than global datasets, under our assumption that national datasets are constructed using better information from direct local knowledge. *Q*_*a*_ was defined as:

2 $${Q}_{a}=\{\begin{array}{cc}1 & {\rm{i}}{\rm{f}}\,{\rm{n}}{\rm{a}}{\rm{t}}{\rm{i}}{\rm{o}}{\rm{n}}{\rm{a}}{\rm{l}}\,{\rm{o}}{\rm{r}}\,{\rm{m}}{\rm{u}}{\rm{l}}{\rm{t}}{\rm{i}}-{\rm{n}}{\rm{a}}{\rm{t}}{\rm{i}}{\rm{o}}{\rm{n}}{\rm{a}}{\rm{l}}\\ 0.5 & {\rm{i}}{\rm{f}}\,{\rm{g}}{\rm{l}}{\rm{o}}{\rm{b}}{\rm{a}}{\rm{l}}.\end{array}$$

*Q*_*s*_ described the primary data source used to develop a dataset. We assumed that datasets developed using survey data (i.e., field survey and censuses) have higher quality than those based on satellite imagery, with datasets constructed using modelling techniques having the lowest quality. We used *Q*_*s*_ to also account for hybrid methods, assigning in such cases intermediate quality scores, as follows:

3 $${Q}_{s}=\left\{\begin{array}{cc}{\rm{1}} & {\rm{survey,}}\,{\rm{satellite,}}\,{\rm{model}}\,{\rm{integration}}\\ {\rm{0}}{\rm{.8}} & {\rm{survey}}\,{\rm{and}}\,{\rm{satellite}}\,{\rm{integration}}\\ {\rm{0}}{\rm{.7}} & {\rm{survey}}\,{\rm{and}}\,{\rm{model}}\,{\rm{integration}}\\ {\rm{0}}{\rm{.5}} & {\rm{satellite}}\,{\rm{and}}\,{\rm{model}}\,{\rm{integration}}\\ {\rm{0}}{\rm{.5}} & {\rm{survey}}\,{\rm{only}}\\ {\rm{0}}{\rm{.3}} & {\rm{satellite}}\,{\rm{only}}\\ {\rm{0}}{\rm{.2}} & {\rm{model}}\,{\rm{only}}\end{array}\right.$$

*Q*_*v*_ was used to rank the level of validation of a dataset, against ground truth, users’ feedback, statistical data, satellite images or other sources. We ranked the validation level from high to low based on the presence of field observations, the number of sources used for validation, and the separation between calibration and validation sets. *Q*_*v*_ was defined as:

4 $${Q}_{v}=\left\{\begin{array}{ll}1, & {\rm{if}}\;{\rm{validated}}\;{\rm{using}}\;{\rm{groundtruth}}\;{\rm{data}}\;{\rm{with}}\;{\rm{sound}}\;{\rm{statistical}}\;{\rm{approaches}}\\ 0.5, & {\rm{if}}\;{\rm{validated}}\;{\rm{using}}\;{\rm{statistics,\; users}}\mbox{'}\;{\rm{feedback}}\;{\rm{or}}\;{\rm{satellite}}\;{\rm{images}}\\ 0, & {\rm{if}}\;{\rm{no}}\;{\rm{attempt}}\;{\rm{of}}\;{\rm{validation}}.\end{array}\right.$$

*Q*_*r*_ described the spatial resolution *r* of a dataset. A higher rank was given to a dataset with finer resolution:

5 $${Q}_{r}={\rm{1}}-\frac{r-{r}_{min}}{{r}_{max}-{r}_{min}},$$

where *r*_*min*_ = 0.0000833° and *r*_*max*_ = 0.0833° were the finest and coarsest resolutions across input datasets.

*Q*_*m*_ was used to assess the level of maturity of a dataset, depending on the frequency of revisions, updates, or releases:

6 $${Q}_{m}=\left\{\begin{array}{ll}1 & {\rm{if}}\,{\rm{annual}}\\ 0.5 & {\rm{if}}\,{\rm{every}}\,{\rm{some}}\,{\rm{years}}\\ 0 & {\rm{if}}\,{\rm{never}}{\rm{.}}\end{array}\right.$$

*Q*_*d*_ was used to assess the level of officiality, i.e., whether a dataset was the result of an official government or non-government dispatch, assuming that official government dispatches have higher reliability than those conducted by non-government entities. It was defined as

7 $${Q}_{d}=\left\{\begin{array}{ll}{\rm{1}} & {\rm{if}}\,{\rm{government}}\\ {\rm{0}}{\rm{.5}} & {\rm{if}}\,\mathrm{non} \mbox{-} \mathrm{government}{\rm{.}}\end{array}\right.$$

All endogenous data quality indicators values are reported in Table 2 below.

**Table 2:** Endogenous dataset quality indicators of all input datasets.

Dataset	Synchrony *Q*_*y*_	Administration *Q*_*a*_	Source *Q*_*s*_	Validation *Q*_*v*_	Resolution *Q*_*r*_	Maturity *Q*_*m*_	Dispatch *Q*_*d*_	${\boldsymbol{\sum }}_{{\boldsymbol{k}}}{{\boldsymbol{Q}}}_{{\boldsymbol{k}}}$
^(a)^MRF	0	0.5	0.5	0.5	0	0	0.5	2
SPAM	0.667	0.5	0.7	0.5	0	0.5	0.5	3.367
GAEZ + 2015	1	0.5	0.5	0.5	0	0	0.5	3
GEOGLAM	0.867	0.5	0.5	0.5	0.400	0	0.5	3.267
OIPA	1	0.5	0.5	1	1.000	0	0.5	4.5
RAP	1	0.5	0.3	0.5	1.000	0	0.5	3.8
EU	1	1	0.8	1	1.000	0	0.5	5.3
SPAMAF	1	1	0.7	0	0.004	0.5	0.5	3.704
AFCAS	0.933	1	0.5	0	0.901	0	0.5	3.834
SASOY	0.933	1	0.8	1	0.998	0	0.5	5.231
MYSTHA	1	1	0.3	0.5	0.998	0	0.5	4.298
ASIARICE	1	1	0.3	0.5	0.947	0	0.5	4.247
CIVGHA	1	1	0.8	1	1.000	0	0.5	5.3
UZBTJK	1	1	0.5	1	1.000	0	0.5	5
USA	0.933	1	0.8	1	0.998	1	1	6.731
CA	1	1	0.8	1	0.998	1	1	6.798
AFG	1	1	0.8	1	1.000	0	0.5	5.3
DEU	1	1	1	0.5	1.000	0	0.5	5
CHNWH	1	1	0.8	1	0.997	0	0.5	5.297
CHNMZ	1	1	0.3	1	0.937	0	0.5	4.737
CHNMZWHRI	1	1	0.3	1	0.941	0	0.5	4.741
BGDRICE	1	1	0.8	1	1.000	0	0.5	5.3
BRA	1	1	0.8	0.5	0.998	0	0.5	4.798
SEN	1	1	0.8	1	1.000	0	0.5	5.3
AU	0.733	1	0.7	0	0.975	0.5	1	4.908
FR	0.933	1	0.3	1	1.000	1	0.5	5.733
JP	1	1	0.8	1	1.000	0.5	1	6.3
CHNMZSOY	0.95	1	0.8	1	1.000	0	0.5	5.25

^(a)^Endogenous qualities in MRF datasets are used for overall data quality but not for multi-criteria selection ranking (Step 4) when other datasets are available for the construction of CROPGRIDS.

### Compute exogenous data quality indicators (Step 3)

Exogenous data quality indicators were defined to describe the quality of a dataset against independent external information. They included *Q*_*CAM*_, comparison against the cropland agreement map (CAM)^18^, and *Q*_*FAO*_, comparison against FAOSTAT harvested area^19^ in the year 2020. Unlike the endogenous indicators, exogenous data quality indicators were evaluated for each input dataset by crop and country.

Specifically, *Q*_*CAM*_ was used to measure the level of agreement of the crop spatial distribution in a dataset against CAM, which is a 2020-updated, statistically robust cropland mask integrating six independent cropland data products. We first converted the *CA* maps of each dataset and the cropland area map of CAM into binary maps, where a grid cell was assigned a value of one for non-zero crop area or zero otherwise. We then calculated *Q*_*CAM*_ for crop *i* in country *j* as:

8 $${Q}_{CAM,i,j}=\frac{{N}_{overlap}(i,j)}{{N}_{CA}(i,j)},$$

where *N*_*CA*_ (*i*, *j*) is the number of grid cells identified as crop *i* in country *j* in a given dataset and *N*_*overlap*_ is the number of grid cells where both CAM and the given dataset have non-zero values.

*Q*_*FAO*_ was used to measure the relative error of the input dataset crop harvested area against FAOSTAT^19^. For crop *i* in country *j*, *Q*_*FAO,i,j*_ was defined as:

9 $${Q}_{FAO,i,j}={\rm{1}}-\mathrm{min}\left\{{\rm{1}},\frac{| HA(i{\rm{,}}j)-{HA}_{FAO}(i{\rm{,}}j)| }{{HA}_{FAO}(i{\rm{,}}j)}\right\}.$$

where *HA*(*i*,*j*) is the total harvested area of crop *i* in country *j* in a dataset, and *HA*_*FAO*_ is the corresponding FAOSTAT value for the year 2020. *Q*_*FAO*_ ranges between 0 and 1, with *Q*_*FAO*_ = 1 representing a perfect match against FAOSTAT. For specific crops where some countries and territories were not included in FAOSTAT, we set *Q*_*FAO*_ = 0.

### Assemblage of global harvested and crop area maps (Step 4)

Assemblage of geo-referenced harvested and crop area maps for individual crops and countries was conducted along two alternative pathways of availability: (1) only MRF data is available; or (2) multiple input datasets are available. In the first case, we proceeded to Step 5 (described later). In the second case, we used the multi-criteria ranking scheme based on endogenous and exogenous data quality indicators described above to select and use data from the dataset with the highest combined quality scores, *Q*_*k,i,j*_, defined in relation to input dataset *k* for crop *i* in country *j* as:

10 $${Q}_{k,i,j}=\frac{1}{3}\times \frac{{\left({Q}_{y}+{Q}_{a}+{Q}_{s}+{Q}_{v}+{Q}_{r}+{Q}_{m}+{Q}_{d}\right)}_{k,i,j}}{{\rm{7}}}+\frac{{Q}_{CAMk,i,j}}{3}+\frac{{Q}_{FAOk,i,j}}{3}.$$

The best-fit datasets *k*_*best*_ for crop *i* in country *j* are provided in Supplementary Table S3. In this case, the MRF dataset was excluded from the ranking. Hence, if only one dataset other than MRF is available, it will be automatically selected as the best dataset.

For each crop, we then compiled an *Arlecchino* map (mosaic) of *HA* and *CA* from best-fit datasets into one global map including all countries. The result of the multi-criteria analysis was that 27 out of the 28 geo-referenced datasets were included in CROPGRIDS (MYSTHA was not selected).

### Data gap filling with NSOs (Step 5)

In the first case of Step 4 when MRF is the only dataset available for a specific country-crop pair, we used NSOs subnational data to update *HA* (see list of available NSO in Supplementary Table S1). Specifically, we spatially disaggregated the subnational-level data following the same principle used in MRF^2^, i.e., by spreading tabulated harvested areas over an assigned agricultural region. For a given crop *i* in subnational unit *j*, we iteratively calculated the harvested area in each grid cell *g* belonging to subnational unit *j* using the CAM percentile *p* as,

11 $$H{A}_{i,g\in j,p}=NS{O}_{i,j}\frac{CA{M}_{g\in j,p}}{{\sum }_{g\in j}CA{M}_{g,p}}$$

with *NSO*_*i,j*_ being the tabulated NSOs values and *CAM*_*g,p*_ being the total cropland area in a grid cell *g* reported by CAM crop mask at *p* percentile. Note that *HA* in Eq. (11) is not uniformly distributed over the subnational unit *j*, but it follows the distribution of the percentile maps in CAM. We used up to 5 percentiles (2.5%, 5%, 10%, 25% and 50%), which correspond to a sequence of maps ranging from the smallest to the median agricultural land area, respectively. We stopped the iteration over the percentiles *p* when we first found some grid cells in which *HA* fell below the lower bound of 100 m^2^. In those grid cells, *HA* was adjusted to 100 m^2^, consistent with the lower bound used in Step 1. The spatialized harvested areas for crop *i* in subnational unit *j* (*HA*_*i,j,g*_) were hence determined. Finally, we calculated $C{A}_{i,g}=\min \left\{H{A}_{i,g},L{A}_{g},CAM9{5}_{g}\right\}$, and we updated *HA*_*i,g*_ = *CA*_*i,g*_ (i.e., we assume *CI* = 1). When NSOs subnational data are not available for a specific country-crop pair, we repeated the spatial information of MRF.

Note that for all datasets, including NSO-updated data, the quality calculated in Eq. (10) is outputted and distributed with this data product in the form of maps (see Table 3). The endogenous quality indicators for NSOs datasets are reported in Supplementary Table S4. However, specifically for NSOs, data quality in Eq. (10) excluded *Q*_*CAM*_ as CAM was used for spatialization.

**Table 3:** CROPGRIDS data distribution files and variables.

Folder Name	File Name	Description	Variable		
			Name	Description	Unit
CROPGRIDSv1.08_NC_maps.zip	CROPGRIDSv1.08_**YYYY**.nc	Contains globally gridded data for crop **YYYY**.	harvarea	Harvested area	hectares
			croparea	Crop (physical) area	hectares
			qual	Data quality	—
			set	Best-fit dataset used	—
	Countries_2018.nc	Contains GAUL^21^ country mask (level 0) used in this study.	country	Country name and code	—
CROPGRIDSv1.08_PNG_maps.zip	CROPGRIDSv1.08_**YYYY**.png	Images of harvested and crop areas of crop **YYYY**, and corresponding data quality and best-fit datasets.			
	Table_CROPGRIDS1.08_COU.xlsx	Excel table of areas for each 173 crops in each country.	Harvested area	Harvested area	hectares
			Crop (physical) area	Crop (physical) area	hectares
CODES.zip	MAIN.m, Aggregate_REMAP.m, Correct_REMAP.m, BUILD_PATCH.m, BUILD_PATCH_fun10.m, ADDNSO_RAW.m, CORRECT_RAWNSO.m, ADJUST_CORRECTED.m	MATLAB scripts used to construct CROPGRIDS (Steps 1 to 6 in Fig. 1).			
	CROPS_matching_v17.xlsx	Excel table of crop name aggregation and matching.			
	DATASET_qualities_v10.xlsx	Excel table showing the endogenous qualities of gridded input datasets.			
	DATASET_NSO_qualities_v1.xlsx	Excel table showing the endogenous qualities of NSOs data.			
	FAOSTAT_ALL_2020_edited.xlsx	National-level harvested areas for year 2020 obtained from FAOSTAT.			

All files are publicly available from *figshare* repository^25^.

### Data adjustment to FAOSTAT 2020 (Step 6)

After the assemblage of georeferenced maps, *HA* of individual crop types in individual countries were scaled to the corresponding country data in FAOSTAT in year 2020^19^. Scaling was performed with an iterative scheme minimizing the distance in harvested area *HA* from FAOSTAT with two constraints - the lower bound (100 m^2^) and the upper bound *LA*. Adjustment of *CA* was conducted simultaneously to *HA* by retaining the crop intensity ratio *CI* in any specific grid cell. When scaled values of $\sum CA$ exceeded *LA* (upper bound), the excess crop area was redistributed uniformly to all other grid cells within that country where each of the individual crops are present. Similarly, when the individual scaled values of *HA* became smaller than 100 m^2^ (lower bound), excess area was uniformly removed from all other grid cells within that country containing that crop. We limited the number of iterations to 60 or when the total crop area adjusted in all crop types in a country was less than 0.5% different than in FAOSTAT in an iteration or when the relative change in total crop area was smaller than 0.01% in an iteration. These thresholds used to limit adjustment iterations were empirically chosen to ensure a balance between data quality and computational efficiency. Adjustment of CROPGRIDS to FAOSTAT was only carried out for all crops and countries that existed in both datasets (153 crops and 185 countries). For a given country-crop pair, a same adjustment factor was applied to all the grid cells containing that crop in that country. Only less than 7% of country-crop pairs required adjustment by a factor smaller than 0.1 or greater than 10 (Supplementary Figure S1). Specifically, the median adjustment factors are not significantly different across crops except for blueberry, mushrooms and triticale, which also show the largest spread across countries (Supplementary Figure S2). Similarly, the median adjustment factors of individual crops are not significantly different across countries, with a few countries showing a large spread (i.e., Afghanistan, Algeria, Iraq, Malta and Qatar, Supplementary Figure S3).

In this step, we simultaneously checked and verified again that the sum of *CA* across all crops is always smaller than or equal to *LA* in each grid cell and that, for individual crops, *CA* ≤ *HA*, and *CI* = *HA/PA* ≤ 3 were always satisfied.

**Fig. 2:**
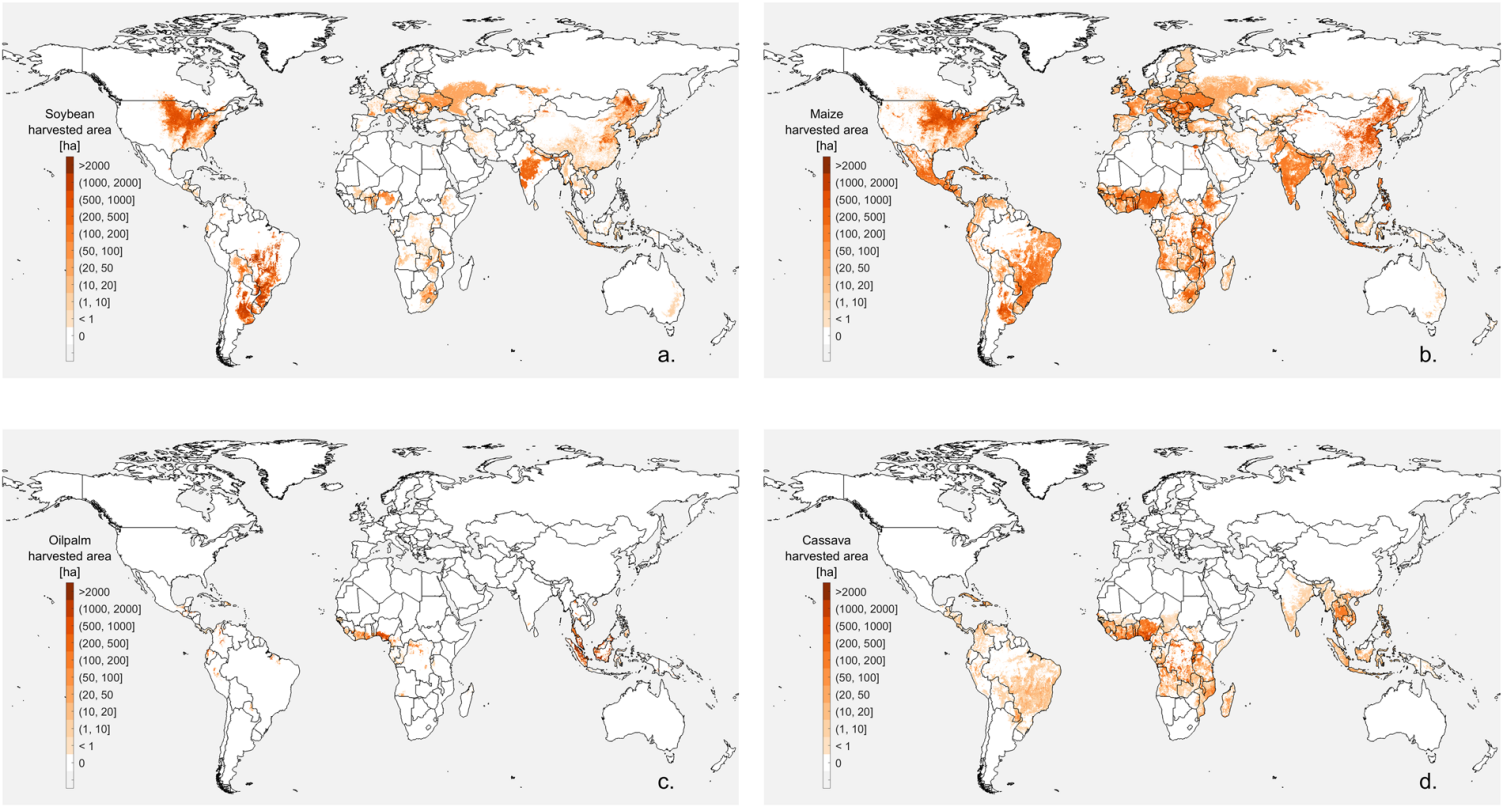
Harvested area maps in CROPGRIDS for the top four crops experiencing the largest expansion since 2000. (**a**) Soybean, (**b**) maize, (**c**) oil palm, and (**d**) cassava.

We presented examples of harvested area maps for the top four crops experiencing major changes since 2000, i.e., oil palm, soybean, cassava, and maize (Fig. 2).

## Data Records

CROPGRIDS dataset distributes global georeferenced maps of harvested and crop (physical) areas and corresponding data quality for 173 crops (refer to Supplementary Table S3 for the list of crops, where coir and gum originally available in MRF are not included in CROPGRIDS) for the year 2020 at a resolution of 0.05° (~5.6 km at the equator) with a bounding box of −180° to 180° longitude and −90° to 90° latitude using the WGS-84 coordinate system. The georeferenced maps are distributed as NetCDF files, which also provides detailed legend of values. This dataset is available for public download from the *figshare* repository^25^ at 10.6084/m9.figshare.22491997. The files included in this distribution are described in Table 3.

## Technical Validation

The majority of the datasets listed in Table 1 included certain level of validation, which are summarized in Supplementary Table S5. Among the 28 input datasets, 17 included validation against ground truth data. In this work, we independently evaluated data on *HA* and *CA* in CROPGRIDS against (1) official national and subnational statistics of crop-specific harvested area (full references provided in Supplementary Table S1); (2) FAOSTAT land areas under temporary and permanent crops by country^26^; and (3) the grid cell-level cropland areas calculated from CAM^4,18^. In addition to independent validations, we conducted uncertainty analysis to test the robustness of the multi-criteria selection ranking and provided comparison of the national-level crop specific *HA* in CROPGRIDS against corresponding FAOSTAT data for 2020, which was used to construct CROPGRIDS.

### Validation of CROPGRIDS with official national and subnational data

We compiled a library of independent datasets of national and subnational harvested area by crop from 36 NSOs (Supplementary Table S1), covering 71 countries and territories and 861 subnational units. Of these, 40 countries and territories reported subnational-level data and 35 reported more than 20 crops each, resulting in a total of 1,852 points available at national-level and 12,149 points at subnational-level. Among the subnational-level data points, 4,832 points were used to construct CROPGRIDS, leaving 7,317 points available for independent validation. In total, evaluations of 106 crop data were conducted against these independent crop statistics from NSOs.

We matched and aggregated crop types in each NSO dataset to match those reported in CROPGRIDS. We used the GAUL^21^ dataset (level 1) to identify subnational units and perform relevant aggregations from pixel level to administrative level 1. The calculations were conducted for 106 crops and were quantified using the coefficient of determination R^2^ (analogue to Nash–Sutcliffe efficiency, NSE^27^) and normalized root mean squared errors (NRMSE) as

12 $${R}_{i}^{2}=1-\frac{{\sum }_{(j)}{\left[{HA}_{NSO}(i,j)-HA(i,j)\right]}^{{\rm{2}}}}{{\sum }_{(j)}{\left[{HA}_{NSO}(i,j)-\overline{{HA}_{NSO}(i,j)}\right]}^{{\rm{2}}}},$$

13 $${{\rm{NRMSE}}}_{i}=\frac{\sqrt{\frac{{\sum }_{(j)}{\left[{HA}_{NSO}\left(i,j\right)-HA\left(i,j\right)\right]}^{{\rm{2}}}}{n}}}{\left[{HA}_{NSO,max}(i)-{HA}_{NSO,min}(i)\right]},$$

where *HA*(*i,j*) and *HA*_*NSO*_(*i,j*) are the harvested area of crop *i* in administrative unit *j* reported by CROPGRIDS and NSOs, respectively, $\overline{{HA}_{NSO}}$ is the average of all NSOs data points, *HA*_*NSO,max*_ and *HA*_*NSO,min*_ are the corresponding maximum and minimum crop harvested areas of NSOs, and *n* is the number of data points.

**Fig. 3:**
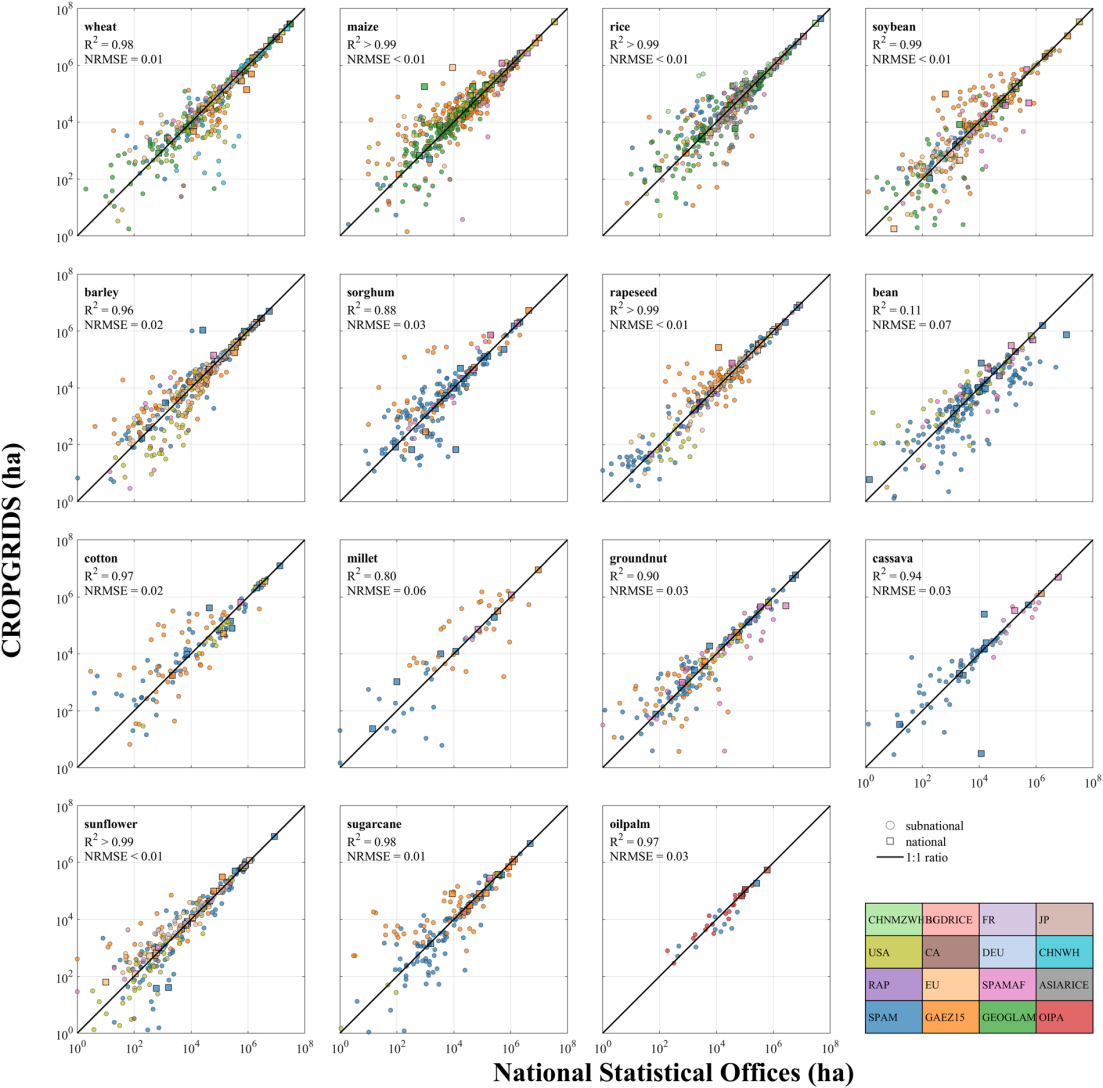
Validation of crop harvested areas in CROPGRIDS against data from National Statistical Offices at national and subnational levels. The colours of the markers refer to the georeferenced datasets selected to use in CROPGRIDS. “Squared” markers represent national-level data, while “circled” markers represent subnational-level data. This figure shows only the validation for the top 15 crops with the largest global harvested area. The validations for the other 91 crops are shown in Supplementary Figure S4.

Among the 106 crops suitable for comparison, the harvested area of 81 crops in CROPGRIDS agreed relatively well with data from NSOs (R^2^ > 0.5, NRMSE < 0.2, Fig. 3 and Supplementary Figure S4). Specifically, the comparisons for important crops such as wheat, maize, rice, soybean, barley, rapeseed, cotton, cassava, sunflower, sugarcane, and oil palm had R^2^ > 0.95 and NRMSE ≤ 0.05, showing very good agreement with officially reported national and subnational statistics (Fig. 3).

### Validation of CROPGRIDS with FAOSTAT land area under temporary and permanent crops

The crop (physical) area in CROPGRIDS refers to FAO land use classes ‘temporary’ or ‘permanent’ crops, depending on crop type^28^. Here, we compared crop areas with FAOSTAT land areas under temporary and permanent crops for 2020 in more than 180 countries. We first classified the 173 crop types included in CROPGRIDS into temporary and permanent crops following the ICC classification^24^ (see Supplementary Table S3 for details). We used the GAUL^21^ dataset (level 0) to identify country boundaries and perform relevant aggregations from pixel level to national level. The goodness of comparison was evaluated using R^2^ and NRMSE as

14 $${R}_{i}^{2}=1-\frac{{\sum }_{(j)}{\left[{CA}_{FAO}\left(i{\rm{,}}j\right)-CA\left(i{\rm{,}}j\right)\right]}^{{\rm{2}}}}{{\sum }_{(j)}{\left[{CA}_{FAO}\left(i{\rm{,}}j\right)-\overline{{CA}_{FAO}\left(i{\rm{,}}j\right)}\right]}^{{\rm{2}}}},$$

15 $${{\rm{NRMSE}}}_{i}=\frac{\sqrt{\frac{{\sum }_{{\rm{(}}j{\rm{)}}}{\left[{CA}_{FAO}\left(i,j\right)-CA\left(i,j\right)\right]}^{{\rm{2}}}}{n}}}{\left[{CA}_{FAO,\max }\left(i\right)-{CA}_{FAO,\min }\left(i\right)\right]},$$

where *CA*(*i,j*) and *CA*_*FAO*_ (*i,j*) are the crop area of either temporary or permanent crops (indicated as *i*) in country *j* reported by CROPGRIDS and FAOSTAT, respectively, $\overline{C{A}_{FAO}}$ is the average of all FAOSTAT data points, *CA*_*FAO,max*_ and *CA*_*FAO,min*_ are the corresponding maximum and minimum temporary or permanent crop areas of FAOSTAT, and *n* is the number of data points.

**Fig. 4:**
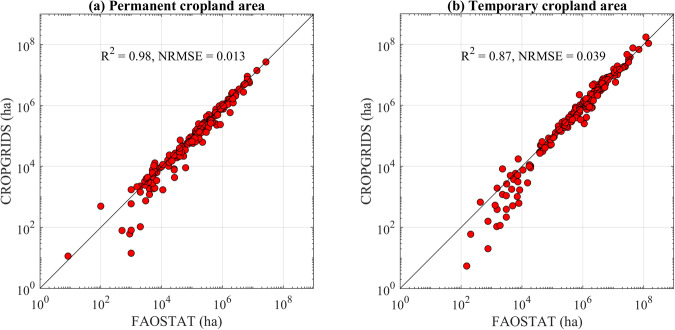
Validation of permanent (**a**) and temporary (**b**) crop (physical) area in CROPGRIDS against FAOSTAT of year 2020. Each circle representing one country.

In CROPGRIDS, the 2020 world total permanent crop area was 167 million ha, consistent with but approximately 8% lower than the 181 million ha reported by FAOSTAT. At national-level, the permanent crop areas determined from CROPGRIDS matched well with values reported by FAOSTAT with R^2^ = 0.98 and NRMSE = 0.01 (Fig. 4a). Additionally, temporary crops in CROPGRIDS covered 1.22 billion ha of global cropland area, and overestimated by approximately 13% the temporary crop area reported in FAOSTAT for 2020, which is 1.08 billion ha. The comparison of temporary crop areas at national-level showed a relatively good match to FAOSTAT data, with R^2^ = 0.87 and NRMSE = 0.04 (Fig. 4b). The overall overestimation of temporary crop area by CROPGRIDS may arise from multiple cropping of different crops, i.e., we may have counted the cropland area more than once if the same piece of land was cultivated with more than one type of crop in a year.

### Comparison of CROPGRIDS crop area with CAM

We next validated the *CA* of all crops included in CROPGRIDS geo-spatially against the cropland area in CAM. Firstly, we calculated the sum of the *CA* of all crops in each grid cell *g* in CROPGRIDS, *CA*_*TOT*_(*g*). Next, for each grid cell *g*, we calculated the 5^th^, $C{A}_{CAM}^{5th}\left(g\right)$, and 95^th^, $C{A}_{CAM}^{95th}\left(g\right)$, percentile cropland area based on CAM dataset^18^. For each grid cell, we then determined if *CA*_*TOT*_(*g*) calculated based on CROPGRIDS falls within $C{A}_{CAM}^{5th}\left(g\right)$ and $C{A}_{CAM}^{95th}\left(g\right)$.

**Fig. 5:**
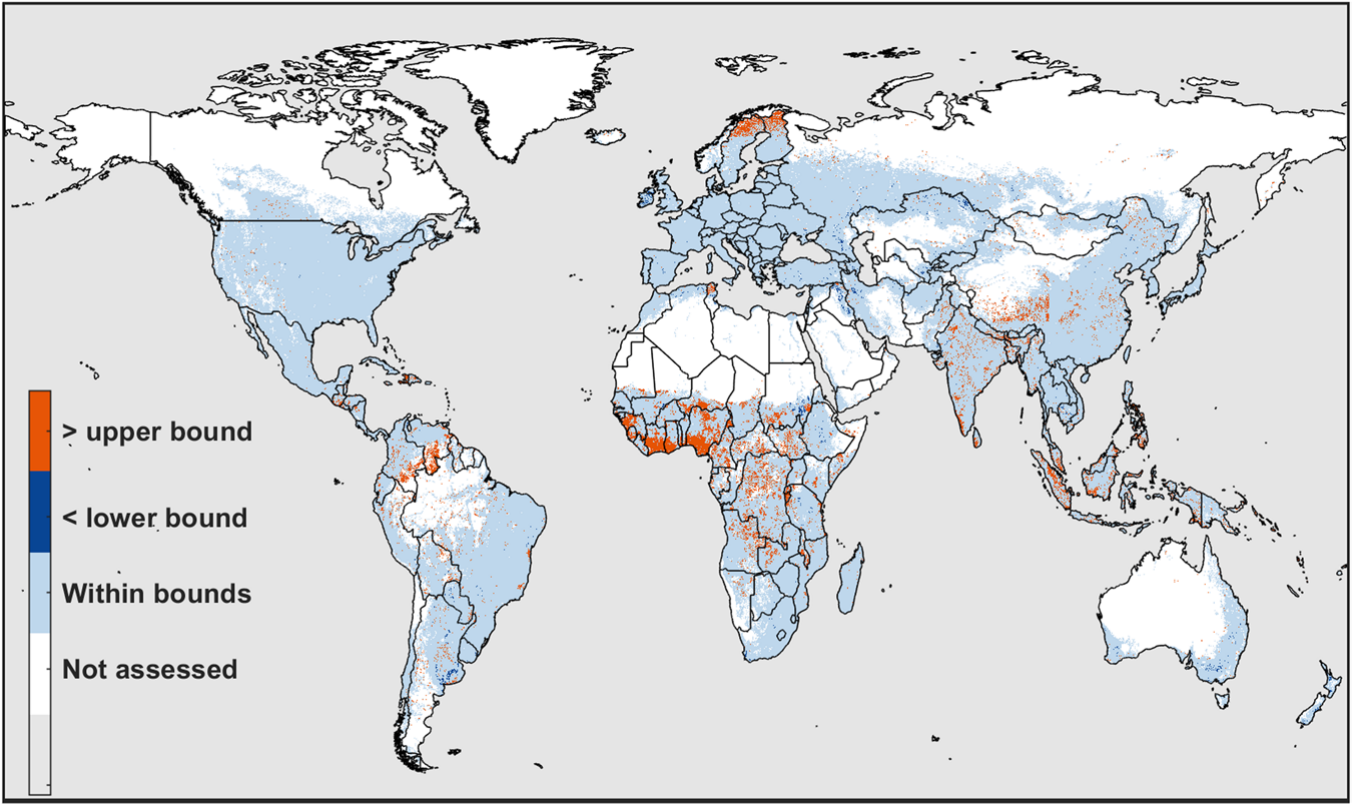
Comparison of the total crop area of all crops included in CROPGRIDS against the cropland area in CAM. The colours in the map illustrate if the total crop area estimated in CROPGRIDS in each grid cell falls within the lower (5th percentile) and upper (95th percentile) bounds of cropland area calculated from CAM.

About 97% of grid cells identified as cropland (i.e., total *CA* across all crops in a grid cell >0) in CROPGRIDS were also identified as cropland in CAM. Globally, the crop areas of about 93% of grid cells identified as cropland in CROPGRIDS fall within $C{A}_{CAM}^{5th}$ and $C{A}_{CAM}^{95th}$, with less than 1% falling below the lower bound (Fig. 5). Those grid cells that have crop area greater than $C{A}_{CAM}^{95th}$ were majorly found in African countries (e.g. Nigeria, Ghana, Cote d’Ivoire) where the six land cover layers used to build CAM are characterized by very high uncertainty.

### Comparison of crop harvested area in CROPGRIDS against FAOSTAT

We compared global-level and national-level crop-specific harvested areas of year 2020 obtained from FAOSTAT^19^ against the corresponding values computed from CROPGRIDS. We used the GAUL^21^ dataset (level 0) to aggregate harvested areas of each crop in CROPGRIDS from pixel level to national level. This comparison does not represent a fully independent validation as these FAOSTAT data were used in Step 6 to adjust the *HA* and *CA* values in CROPGRIDS. Rather, this comparison serves to provide an estimation of percent error (%∆) between FAOSTAT and CROPGRIDS at national-level, determined as

16 $$ \% \Delta \left(i,j\right)=\frac{HA\left(i,j\right)-H{A}_{FAO}\left(i,j\right)}{H{A}_{FAO}\left(i,j\right)}\times 100$$

where *HA*(*i*,*j*) is the harvested area of crop *i* in country *j* in CROPGRIDS and *HA*_*FAO*_ is the corresponding 2020 FAOSTAT value.

**Fig. 6:**
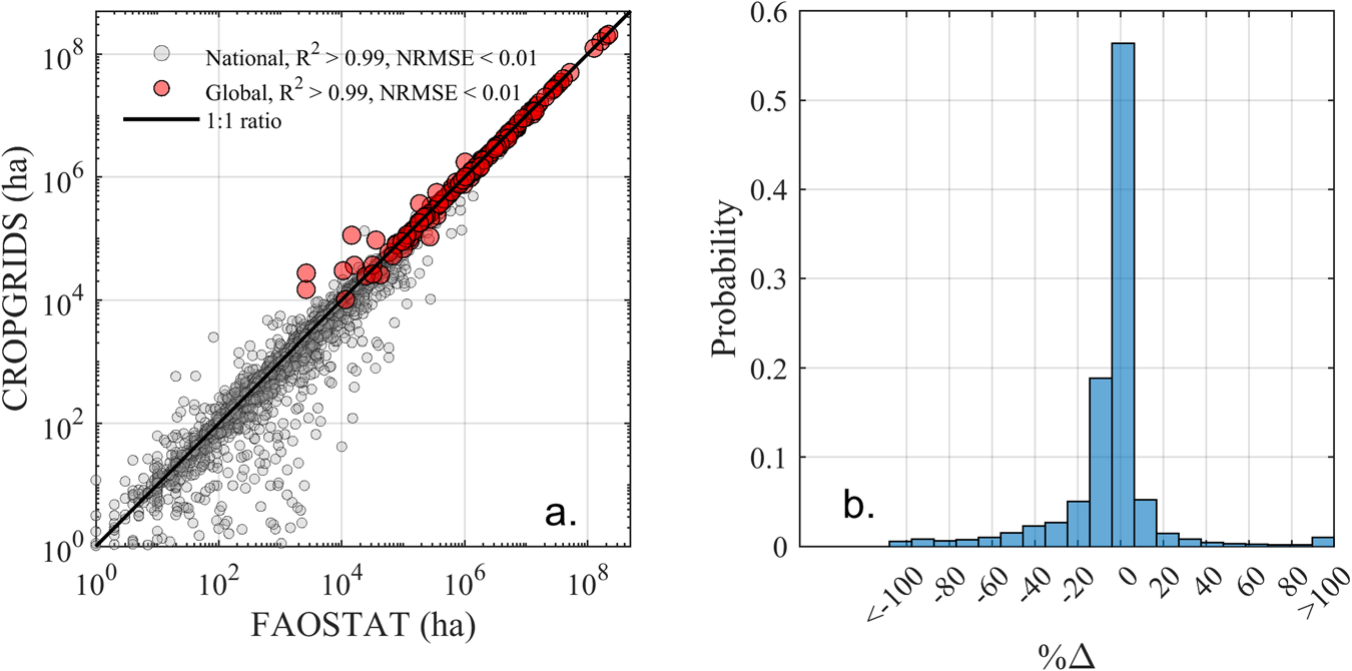
Comparison of crop harvested area in CROPGRIDS with FAOSTAT values for 2020. (**a**) scatter-plot between harvested areas in CROPGRIDS against FAOSTAT, and (**b**) probability distribution of percent error %∆. In total, there were 7,697 pairs of comparisons at national-level and 153 pairs for global crop-specific harvested areas.

The global crop-specific harvested area in CROPGRIDS matched well with those reported in FAOSTAT with an R^2^ ≈ 1.00 and NRMSE < 0.01 (Fig. 6, red markers), with 115 crops having a difference less than ±10% (Supplementary Figure S5). Comparison of CROPGRIDS against national-level crop-specific harvested areas of FAOSTAT also shows good matching with an R^2^ ≈ 1.00 and NRMSE < 0.01 (Fig. 6a, grey markers). About 84% of data points (out of a total of 7,697 pairs) had differences less than ±20%, while only less than 1% had a difference greater than ±100% (Fig. 6b).

### Uncertainty in the multi-criteria selection ranking

The endogenous and exogenous data quality indicators used in the multi-criteria selection were assigned meaningfully but with arbitrary values. We conducted a Monte-Carlo analysis to quantify the uncertainty associated to such arbitrary choices, by introducing random weights, in the range 0–1, to each data quality indicator, that is: {*w*_*c*_, *w*_*a*_, *w*_*s*_, *w*_*v*_, *w*_*r*_, *w*_*m*_, *w*_*d*_} for endogenous and {*w*_*C*_, *w*_*F*_} for exogenous indicators—whereas we implicitly had used unity weights in Eq. (10). We extracted 10,000 values of each of the nine weights from independent Gaussian probability distribution functions with a mean equal to 1 and a standard deviation equal to 0.1 and we limited their values within the range between 0.7 and 1.3, that is three times the standard deviation. We next counted the frequency of occurrence of a selected dataset different than when using the default weight values. This uncertainty analysis was only conducted for combinations of countries and crops where more than one dataset was available.

Results of the Monte-Carlo analysis on the endogenous and exogenous characteristics of the multi-criteria selection ranking scheme suggested that the method of best-fit dataset selection was highly robust. Specifically, for the 78 crops and 187 countries with multiple datasets, the probability that the selection of the best-fit dataset would change with randomized characteristics was highly unlikely (white tiles in Supplementary Figure S6). In a minor fraction of crops and countries, the probability was greater than 10%, with only 40 out of 3352 assessed pairs of crops and countries having a probability ≥40% and only 3 pairs having a probability ≥50% (Supplementary Figure S6).

### Known limitations and uncertainties

CROPGRIDS inherits uncertainties and errors embedded in the input datasets and these uncertainties can stem from a variety of sources. Datasets constructed based on censuses surveys (e.g., MRF and SPAM) can have uncertainties stemming from the methods used to spatialize crop area statistics at administrative-level 2 and the imperfection in statistical reporting of harvested and crop areas. Datasets constructed using remote sensing approaches can suffer from the inherent uncertainties in remote sensing data, such as, atmospheric interference and limitations in spatial resolution. More generally, these datasets also carry forward uncertainties underlying in the cropland layer maps used as their input and can be limited by the availability of ground truth data in certain regions for validation purposes. These uncertainties can propagate through the mapping process and affect the accuracy of the resulting harvested and crop area estimates in CROPGRIDS.

In addition to inherited uncertainties, the construction of CROPGRIDS also suffers from known limitations. Firstly, the imputation of *CA* for temporary crops by taking the minimum value between *HA*, *LA*, and *CAM95* as described in Methods may lead to an overestimation of *CA*, particularly for those crops that undergo multiple harvests. While we have accounted for cropping intensities greater than 1 for crops with multiple harvests (e.g., rice), we have not explicitly accounted for dual and multi-layered cropping systems when more than one crop are grown in the same cultivated area. Information about dual cropping systems across the available datasets is limited, with only the datasets for USA and Canada providing this information. Information on multi-layered cropping systems (e.g., barley below olive trees in some Mediterranean systems or coffee plantations under natural trees) is entirely lacking. The lack of information on cropping practices and irrigation management may contribute uncertainties to crop distribution mapping, potentially leading to both underestimations and overestimations in *HA* and *CA* for some countries and some crops, leaving a knowledge gap that may be filled in future releases of CROPGRIDS. In addition, we did not account explicitly for protected agriculture. The estimation of *LA* excluded urbanization area, which may encompass protected agriculture, and hence, may lead to underestimation of *HA* and *CA* of some horticultural crops. These knowledge gaps highlight the importance of expanding the spatial coverage and frequency of ground monitoring and data collection for agricultural practices to enhance crop distribution mapping.

The approach employed in developing CROPGRIDS involves the integration of crop area data from various years, with the majority of the datasets used having reference years between 2015 and 2020. However, this approach can introduce uncertainties due to potential annual variations in the crop types cultivated in specific regions, influenced by factors such as climatic suitability and market demand.

At a spatial resolution of 0.05°, a grid cell has a size of approximately 5.6 km × 5.6 km, corresponding to about 3000 ha. This leads to uncertainties in the estimated harvested and crop areas for some crops typically cultivated at smaller scales except under intensively managed systems, often monocultures. It furthermore creates uncertainty at the border between two countries and affects in particularly the calculation of the exogenous data quality indicator *Q*_*FAO*_ that compares a dataset against national-level crop harvested area reported by FAOSTAT. This border effect impacts estimates mostly in small countries in two ways. The first is when a country has zero harvested and crop areas for a crop across all datasets because border grid cells fall in the neighbouring country, whereas FAOSTAT reports non-zero values. In this case, no selection is performed. The second is when, in contrast, a country has a harvested area greater than zero when grid cells of other neighbour countries fall within a country and FAOSTAT returns zero value. In this case, datasets will still be ranked and the best-fit will be selected according to other quality indicators. This known bias is difficult to detect and correct, especially for small countries, because whether a border grid cell belongs to one or another country cannot be estimated correctly at the given resolution. Specifically, this bias is scale-dependent and its occurrence decreases with increasing resolution and data quality, including of the layer of administrative boundaries used to extract country statistics. Due to constraint in spatial resolution, CROPGRIDS excludes a few small countries and territories (i.e., Falkland, Faroe Islands, French S.A.T., Heart Island, Isle of Man, Kingman Reef, Kiribati, Ma’tan al-Sarra, Mayotte, Nether. Antilles, Palau, Réunion, Saint Pierre, South Georgia, Svalbard, Virgin Islands). Greenland is also excluded, considering the small area of cultivated land.

Furthermore, uncertainties can also arise from the type of validation data, which themselves may inherit uncertainties. For example, statistics at the subnational level may be masked due to confidentiality issues, especially in cases where significant producers are located in relatively small counties or districts. As a result, the aggregated data at the national level may differ from the data available at the more detailed subnational level, thereby posing limitations in the validation process.

## Usage Notes

All georeferenced maps distributed in CROPGRIDS dataset^25^ are formatted as standard NetCDF4 files, which can be read in various coding languages (e.g., MATLAB, Python, Julia) and software (ArcGIS, QGIS, Panoply). CROPGRIDS dataset also contains a country mask based on GAUL^21^ dataset at administrative level 0, which can be used to aggregate pixel-level data to national-level for comparison against official statistics (e.g., FAOSTAT or other National Statistical Offices). Crop type name in CROPGRIDS follows the naming system used by FAO^19^, allowing direct comparison against FAOSTAT data.

### Supplementary information

Supplementary Information

## Data Availability

All data processing and testing described in Methods and Technical Validation sections were conducted using MATLAB version R2021a. Main codes used to construct CROPGRIDS are distributed in the “CODES.zip” folder (Table 3) along with CROPGRIDS dataset available for public download from the *figshare* repository^25^ at 10.6084/m9.figshare.22491997.
