# Supplementary material for: CROPGRIDS: a global geo-referenced dataset of 173 crops
Source: Sci Data. 2024 Apr 22;11:413. doi: 10.1038/s41597-024-03247-7 (PMC11035692; doi:10.1038/s41597-024-03247-7)
Supplement: Supplementary file 1 — Supplementary Information [file 41597_2024_3247_MOESM1_ESM.pdf]

Supplementary Information

CROPGRIDS: a global geo-referenced dataset of 173 crops

Fiona H. M. Tang[1,2], Thu Ha Nguyen[3], Giulia Conchedda[4], Leon Casse[4], Francesco N. Tubiello[4], Federico Maggi[3,5]

Affiliations:
[1]School of Environmental and Rural Science, University of New England, Armidale, New South Wales 2351, Australia.
[2]Department of Civil Engineering, Monash University, Clayton 3800, Victoria, Australia.
[3]Environmental Engineering, School of Civil Engineering, The University of Sydney, Sydney, New South Wales, Australia.
[4]Statistics Division, Food and Agriculture Organization of the United Nations, Viale delle Terme di Caracalla, Rome 00153, Italy.
[5]Sydney Institute of Agriculture, The University of Sydney, Sydney, NSW, 2006, Australia

Correspondence to: Federico Maggi (federico.maggi@sydney.edu.au)

Table of Contents:

**Supplementary Tables**	
Table S1 Descriptions of crop harvested area data obtained from various national statistical offices.	2 – 7
Table S2 Matching and aggregation of crop names for the 27 input datasets providing georeferenced crop-specific information and the FAOSTAT dataset.	8 – 9
Table S3 The percent of grid cells and countries being updated since 2000 and the selected best-fit datasets used to construct the global maps of harvested and crop areas of each individual crop.	10 – 13
Table S4 Endogenous dataset quality indicators of national statistical offices (NSO) datasets.	14 – 15
Table S5 Summary of dataset validation.	16

**Supplementary Figures**	
Figure S1 The "after-to-before" ratio used in Step 6 to adjust harvested area for matching national-level FAOSTAT data for 2020.	17 – 18
Figure S2 Representation of full statistics of the "after-to-before" ratio used in Step 6 to adjust harvested area for matching national-level FAOSTAT data for 2020 by countries.	19
Figure S3 Representation of full statistics of the "after-to-before" ratio used in Step 6 to adjust harvested area for matching national-level FAOSTAT data for 2020 by crops.	20
Figure S4 Comparison of crop harvested areas at national and subnational levels between CROPGRIDS and National Statistical Offices.	21– 26
Figure S5 Relative differences of global harvested area between CROPGRIDS and FAOSTAT for 153 crops.	27
Figure S6 Uncertainty analysis of the multi-criteria ranking scheme.	28

**References**	29 – 30

Supplementary Tables

Table S1: Descriptions of crop harvested area data obtained from various national statistical offices (NSOs). Note that aggregated values at national level may differ from detailed values at subnational level even though both were obtained from the same data source because some subnational statistics may be masked due to confidentiality issues. When harvested area is not available, we assume that harvested area is equal to planted area.

Country/ Territory name	Number of subnational units	Number of crops	Crops	Year	Data link
Albania	0	14	alfalfa, barley, grape, greencorn, maize, oats, olive, potato, pulsenes, rye, soybean, sugarbeet, sunflower, wheat	2020	https://ec.europa.eu/eurostat/web/agriculture/data/database
Algeria	43	25	alfalfa, artichoke, barley, bean, carrot, chickpea, date, fig, grape, grapefruitetc, greenbean, lemonlime, lentil, maize, oats, olive, onion, orange, pea, potato, sorghum, tangetc, tobacco, tomato, wheat	2015	https://www.ons.dz/spip.php?rubrique379
Argentina	12	7	barley, canaryseed, cotton, oats, pea, rice, safflower	2020	https://datosestimaciones.magyp.gob.ar/reportes.php?reporte=Estimaciones
Armenia	0	29	barley, bean, beetfor, cabbage, carrot, cauliflower, chilleetc, cucumberetc, eggplant, flax, garlic, grape, greenbean, greenpea, lentil, maize, maizefor, melonetc, melonseed, oats, onion, pea, potato, sugarbeet, sunflower, tobacco, tomato, walnut, wheat	2020	https://www.armstat.am/en/?nid=82&year=2022
Australia	8	26	banana, barley, cabbage, carrot, cauliflower, chickpea, cotton, grape, greenbean, greencorn, lentil, lettuce, lupin, maize, melonetc, mushroom, oats, onion, pineapple, pumpkinetc, rapeseed, rice, sorghum, strawberry, sugarcane, wheat	2020	https://www.abs.gov.au/statistics/industry/agriculture/agricultural-commodities-australia
Austria	9	24	alfalfa, barley, broadbean, grape, greencorn, hemp, hop, linseed, lupin, maize, oats, oilseednes, pea, potato, pulsenes, rapeseed, rootnes, rye, sorghum, soybean, sugarbeet, sunflower, triticale, wheat	2020	https://ec.europa.eu/eurostat/web/agriculture/data/database
Azerbaijan	10	52	almond, apple, apricot, barley, bean, cabbage, carrot, cauliflower, cherry, chestnut, cotton, cranberry, cucumberetc, eggplant, fig, garlic, grape, greenpea, hazelnut, kiwi, lemonlime, lentil, maize, melonetc, millet, oats, olive, onion, orange, pea, peachetc, pear, persimmon, pistachio, plum, potato, pumpkinetc, quince, rice, rye, sorghum, sourcherry, sugarbeet, sunflower, tangetc, tea, tobacco, tomato, vetch, walnut, watermelon, wheat	2020	https://stat.gov.az/source/agriculture/
Bangladesh	7	170	abaca, agave, alfalfa, almond, aniseetc, apple, apricot, areca, artichoke, asparagus, avocado, bambara, barley, bean, beetfor, berrynes, blueberry, brazil, broadbean, buckwheat, cabbage, cabbagefor, canaryseed, carob, carrot, carrotfor, cashew, cashewapple, cassava, castor, cauliflower, cerealnes, cherry, chestnut, chickpea, chicory, chilleetc, cinnamon, citrusnes, clove, clover, cocoa, coconut, coffee, coir, cowpea, cranberry, cucumberetc, currant, eggplant, fibrenes, fig, flax, fonio, fornes, fruitnes, garlic, ginger, gooseberry, grape, grapefruitetc, grassnes, greenbean, greenbroadbean, greencorn, greenonion, greenpea, gums, hazelnut, hemp, hempseed, hop, jutelikefiber, kapokfiber, kapokseed, karite, kiwi, kolanut, legumenes, lemonlime, lentil, lettuce, linseed, lupin, maize, maizefor, mango, mate, melonetc, melonseed, millet, mixedgrain, mixedgrass, mushroom, mustard, nutmeg, nutnes, oats, oilpalm, oilseedfor, oilseednes, okra, olive, onion, orange, papaya, pea, peachetc, pear, pepper, peppermint,	2020	http://www.bbs.gov.bd/site/page/453af260-6aea-4331-b4a5-7b66fe63ba61/-

			persimmon, pigeonpea, pimento, pineapple, pistachio, plantain, plum, popcorn, poppy, potato, pulsenes, pumpkinetc, pyrethrum, quince, quinoa, ramie, rapeseed, rasberry, rice, rootnes, rubber, rye, ryefor, safflower, sesame, sisal, sorghum, sorghumfor, sourcherry, soybean, spicenes, spinach, stonefruitnes, strawberry, stringbean, sugarbeet, sugarcane, sugarnes, sunflower, swedefor, sweetpotato, tangetc, taro, tea, tobacco, tomato, triticale, tropicalnes, tung, turnipfor, vanilla, vegetablenes, vegfor, vetch, walnut, watermelon, wheat, yam, yautia		
Belarus	6	11	barley, buckwheat, flax, maize, oats, potato, rapeseed, rye, sugarbeet, triticale, wheat	2020	http://dataportal.belstat.gov.by/Indicators/Preview?key=292487#
Belgium	2	22	alfalfa, barley, broadbean, flax, grape, greencorn, hemp, hop, linseed, maize, oats, oilseednes, pea, potato, pulsenes, rapeseed, rootnes, rye, sugarbeet, tobacco, triticale, wheat	2020	https://ec.europa.eu/eurostat/web/agriculture/data/database
Bosnia and Herzegovina	0	22	alfalfa, barley, bean, beetfor, cabbage, carrot, chilleetc, clover, cucumberetc, garlic, maize, maizefor, oats, onion, pea, potato, rye, soybean, tobacco, tomato, triticale, wheat	2020	http://fzs.ba/index.php/publikacije/saopcenjapriopcenja/poljoprivreda-i-ribarstvo/
Botswana	10	7	groundnut, maize, melonetc, millet, sorghum, sunflower, watermelon	2019	https://www.statsbots.org.bw/latest-publications?title=&field_keywords_value=&field_release_date_value%5Bmin%5D%5Bdate%5D=&field_release_date_value%5Bmax%5D%5Bdate%5D=&field_sector_nid=43
Brunei	0	1	rice	2020	http://www.agriculture.gov.bn/SitePages/Agriculture%20and%20Agrifood's%20Statistic.aspx
Bulgaria	0	21	alfalfa, barley, grape, greencorn, hemp, maize, oats, oilseednes, pea, potato, pulsenes, rapeseed, rice, rootnes, rye, sorghum, soybean, sunflower, tobacco, triticale, wheat	2020	https://ec.europa.eu/eurostat/web/agriculture/data/database
Burkina Faso	0	13	bambara, cotton, cowpea, fonio, groundnut, maize, millet, potato, rice, sesame, sorghum, soybean, yam	2020	http://www.cns.bf/basecns/cns/index.php
Cambodia	23	14	banana, cashew, cassava, coconut, maize, mango, orange, pineapple, rice, rubber, sesame, soybean, sugarcane, sweetpotato	2019	http://nis.gov.kh/index.php/en/about/31-cas/110-cas2020
Cameroon	5	18	bambara, banana, bean, cassava, cowpea, groundnut, maize, millet, okra, onion, plantain, potato, rice, sorghum, soybean, taro, tomato, yam	2019	https://www.minader.cm/index.php/2022/03/22/assessment-of-the-2019-2020-crop-year-and-food-availability-in-the-adamawa-east-far-north-north-and-west-regions/
Canada	10	55	apple, apricot, asparagus, barley, bean, blueberry, buckwheat, cabbage, canaryseed, carrot, cauliflower, cherry, chickpea, chilleetc, cranberry, cucumberetc, currant, eggplant, flax, garlic, grape, greenbean, greencorn, greenonion, greenpea, hemp, lentil, lettuce, maize, maizefor, melonetc, mixedgrain, mustard, oats, onion, pea, peachetc, pear, plum, potato, pumpkinetc, rapeseed, rasberry, rye, sourcherry, soybean, spinach, strawberry, sugarbeet, sunflower, sweetpotato, tomato, triticale, watermelon, wheat	2020	https://www150.statcan.gc.ca/t1/tbl1/en/
Chile	13	65	almond, apple, apricot, artichoke, asparagus, avocado, barley, bean, blueberry, cabbage, carrot, cauliflower, cherry, chestnut, chickpea, chicory, chilleetc, cranberry, cucumberetc, date, fig, garlic, grape, grapefruitetc, greenbean, greencorn, greenonion, greenpea, hazelnut, kiwi, lemonlime, lentil, lettuce, lupin, maize, mango, melonetc, oats, olive, onion, orange, papaya, peachetc, pear, persimmon, pimento, pistachio, plum, potato,	2020	https://www.odepa.gob.cl/estadisticas-del-sector/estadisticas-productivas

			pumpkinetc, quince, rapeseed, rasberry, rice, sourcherry, spinach, strawberry, sugarbeet, tangetc, tobacco, tomato, triticale, walnut, watermelon, wheat		
China	31	10	bean, cotton, groundnut, rapeseed, rice, sugarbeet, sugarcane, tea, tobacco, wheat	2020	http://www.stats.gov.cn/tjsj/ndsj/2021/indexeh.htm
Colombia	31	64	agave, apple, asparagus, avocado, banana, barley, bean, blueberry, broadbean, cabbage, carrot, cashew, cauliflower, chickpea, chilleetc, cocoa, coconut, coffee, cotton, cucumberetc, date, eggplant, fig, garlic, ginger, grape, grapefruitetc, greenbean, greenonion, groundnut, lemonlime, lettuce, maize, mango, melonetc, oats, oilpalm, onion, orange, papaya, pea, peachetc, pear, peppermint, pimento, plantain, plum, potato, pumpkinetc, quinoa, rapeseed, rice, sesame, soybean, spinach, strawberry, sugarcane, sweetpotato, tangetc, tea, tobacco, tomato, wheat, yam	2020	https://www.agronet.gov.co/estadistica/Paginas/home.aspx?cod=1
Costa Rica	7	12	bean, coffee, maize, melonetc, oilpalm, onion, orange, pineapple, potato, rice, sugarcane, watermelon	2020	http://www.sepsa.go.cr/productos_BEA.html
Croatia	0	21	alfalfa, barley, broadbean, grape, greencorn, maize, oats, oilseednes, olive, pea, potato, pulsenes, rapeseed, rootnes, rye, soybean, sugarbeet, sunflower, tobacco, triticale, wheat	2020	https://ec.europa.eu/eurostat/web/agriculture/data/database
Cyprus	0	13	alfalfa, barley, broadbean, grape, greencorn, oats, oilseednes, olive, pea, potato, pulsenes, triticale, wheat	2020	https://ec.europa.eu/eurostat/web/agriculture/data/database
Czech Republic	5	23	alfalfa, barley, broadbean, grape, greencorn, hemp, hop, linseed, lupin, maize, oats, oilseednes, pea, potato, pulsenes, rapeseed, rootnes, rye, soybean, sugarbeet, sunflower, triticale, wheat	2020	https://ec.europa.eu/eurostat/web/agriculture/data/database
D.R. Congo	10	9	bean, cassava, groundnut, maize, potato, rice, soybean, taro, yam	2018	https://minagri.gouv.cd/publications/
Denmark	0	14	alfalfa, barley, broadbean, greencorn, maize, oats, pea, potato, rapeseed, rootnes, rye, sugarbeet, triticale, wheat	2020	https://ec.europa.eu/eurostat/web/agriculture/data/database
Dominican Republic	0	39	avocado, banana, bean, cabbage, carrot, cassava, cauliflower, cherry, chilleetc, coconut, cucumberetc, eggplant, garlic, grapefruitetc, groundnut, lemonlime, lettuce, maize, mango, melonetc, okra, onion, orange, papaya, pigeonpea, pineapple, plantain, potato, pumpkinetc, rice, sorghum, stringbean, sweetpotato, tangetc, tobacco, tomato, watermelon, yam, yautia	2020	https://www.one.gob.do/datos-y-estadisticas/temas/estadisticas-economicas/estadisticas-sectoriales/agricultura-ganaderia-silvicultura-y-pesca/
Ecuador	23	25	avocado, bean, broadbean, cassava, cocoa, coffee, greenonion, greenpea, groundnut, lemonlime, maize, mango, oilpalm, orange, pea, pineapple, plantain, potato, quinoa, rice, soybean, sugarcane, tobacco, tomato, wheat	2020	https://www.ecuadorencifras.gob.ec/encuesta-de-superficie-y-produccion-agropecuaria-continua-2020/
Egypt	27	63	alfalfa, almond, apple, apricot, artichoke, banana, barley, bean, broadbean, cabbage, cauliflower, cherry, chickpea, chilleetc, clover, cotton, cucumberetc, date, eggplant, fig, flax, garlic, grape, grapefruitetc, greenbean, greenbroadbean, greenonion, greenpea, groundnut, lemonlime, lentil, lupin, maize, mango, melonetc, okra, olive, onion, orange, pea, peachetc, pear, pepper, persimmon, pistachio, plum, potato, pumpkinetc, rice, sesame, sorghum, soybean, spinach, strawberry, sugarbeet, sugarcane, sunflower, sweetpotato, tangetc, taro, tomato, watermelon, wheat	2017	https://censusinfo.capmas.gov.eg/Metadata-en-v4.2/index.php/catalog/399
Estonia	0	14	barley, broadbean, greencorn, linseed, oats, oilseednes, pea, potato, pulsenes, rapeseed, rootnes, rye, triticale, wheat	2020	https://ec.europa.eu/eurostat/web/agriculture/data/database
FYR Macedonia	0	15	alfalfa, barley, broadbean, grape, greencorn, maize, oats, potato, rapeseed, rice, rootnes, rye, sunflower, tobacco, wheat	2020	https://ec.europa.eu/eurostat/web/agriculture/data/database
Finland	0	10	barley, broadbean, linseed, oats, pea, potato, rapeseed, rye, sugarbeet, wheat	2020	https://ec.europa.eu/eurostat/web/agriculture/data/database
France	22	27	barley, broadbean, flax, grape, greencorn, hemp,	2020	https://ec.europa.eu/eu

			hop, linseed, lupin, maize, oats, oilseednes, olive, pea, potato, pulsenes, rapeseed, rice, rootnes, rye, sorghum, soybean, sugarbeet, sunflower, tobacco, triticale, wheat		rostat/web/agriculture/data/database
Germany	16	21	barley, broadbean, greencorn, hemp, hop, linseed, lupin, maize, oats, oilseednes, pea, potato, pulsenes, rapeseed, rootnes, soybean, sugarbeet, sunflower, tobacco, triticale, wheat	2020	https://ec.europa.eu/eurostat/web/agriculture/data/database
Greece	13	25	alfalfa, barley, broadbean, grape, greencorn, hemp, linseed, lupin, maize, oats, oilseednes, olive, pea, potato, pulsenes, rapeseed, rice, rye, sorghum, soybean, sugarbeet, sunflower, tobacco, triticale, wheat	2020	https://ec.europa.eu/eurostat/web/agriculture/data/database
Guam	0	1	banana	2020	https://www.fsa.usda.gov/news-room/efoia/electronic-reading-room/frequently-requested-information/crop-acreage-data/index
Hungary	0	24	alfalfa, barley, broadbean, grape, greencorn, linseed, lupin, maize, oats, oilseednes, pea, potato, pulsenes, rapeseed, rice, rootnes, rye, sorghum, soybean, sugarbeet, sunflower, tobacco, triticale, wheat	2020	https://ec.europa.eu/eurostat/web/agriculture/data/database
Iceland	0	4	barley, potato, rapeseed, rootnes	2020	https://ec.europa.eu/eurostat/web/agriculture/data/database
India	31	19	barley, castor, chickpea, cotton, groundnut, jute, lentil, linseed, maize, millet, pigeonpea, rice, safflower, sesame, sorghum, soybean, sugarcane, sunflower, wheat	2020	https://eands.dacnet.nic.in/APY_96_To_06.htm
Iran	28	3	barley, rice, wheat	2014	https://irandataportal.syr.edu/socio-economic-data/statistical-yearbook
Ireland	0	13	alfalfa, barley, broadbean, greencorn, hemp, linseed, oats, pea, potato, rapeseed, rootnes, sunflower, wheat	2020	https://ec.europa.eu/eurostat/web/agriculture/data/database
Italy	20	27	alfalfa, barley, broadbean, flax, grape, greencorn, hemp, hop, lupin, maize, oats, oilseednes, olive, pea, potato, pulsenes, rapeseed, rice, rootnes, rye, sorghum, soybean, sugarbeet, sunflower, tobacco, triticale, wheat	2020	https://ec.europa.eu/eurostat/web/agriculture/data/database
Japan	47	29	apple, apricot, barley, buckwheat, cabbage, carrot, chilleetc, cucumberetc, eggplant, grape, greenonion, lettuce, melonetc, onion, peachetc, pear, potato, pumpkinetc, rapeseed, rice, soybean, spinach, strawberry, sugarbeet, sugarcane, sweetpotato, taro, tea, tomato	2020	https://www.stat.go.jp/english/data/nenkan/72nenkan/1431-08.html
Laos	16	9	coffee, cotton, groundnut, maize, rice, soybean, sugarcane, tea, tobacco	2020: maize, coffee; 2019 for others	https://laosis.lsb.gov.la/tblInfo/TblInfoList.do
Latvia	0	17	alfalfa, barley, broadbean, flax, greencorn, linseed, lupin, oats, oilseednes, pea, potato, pulsenes, rapeseed, rootnes, rye, triticale, wheat	2020	https://ec.europa.eu/eurostat/web/agriculture/data/database
Lithuania	0	20	alfalfa, barley, broadbean, greencorn, hemp, linseed, lupin, maize, oats, oilseednes, pea, potato, pulsenes, rapeseed, rootnes, rye, soybean, sugarbeet, triticale, wheat	2020	https://ec.europa.eu/eurostat/web/agriculture/data/database
Luxembourg	0	17	barley, broadbean, grape, greencorn, maize, oats, oilseednes, pea, potato, pulsenes, rapeseed, rootnes, rye, soybean, sunflower, triticale, wheat	2020	https://ec.europa.eu/eurostat/web/agriculture/data/database
Malaysia	13	28	banana, cabbage, carrot, cassava, cauliflower, coconut, coffee, cucumberetc, ginger, greencorn, groundnut, lemonlime, lettuce, mushroom, mustard, nutmeg, okra, papaya, pineapple, pumpkinetc, rice, spinach, sugarcane, sweetpotato, tea, tomato, watermelon, yam	2020	https://www.mafi.gov.my/penerbitan
Malta	0	2	grape, potato	2020	https://ec.europa.eu/eurostat/web/agriculture/data/database
Mexico	32	91	agave, alfalfa, aniseetc, apple, apricot, artichoke,	2018	https://nube.siap.gob.

			asparagus, avocado, barley, bean, blueberry, broadbean, cabbage, canaryseed, carob, carrot, cashew, cassava, cauliflower, cherry, chickpea, clover, cocoa, coconut, coffee, cotton, date, eggplant, fig, garlic, ginger, grape, grapefruitetc, greenbean, greenbroadbean, greencorn, groundnut, lemonlime, lentil, lettuce, linseed, maize, maizefor, mango, melonetc, millet, mushroom, oats, oilpalm, okra, olive, onion, orange, papaya, pea, peachetc, pear, peppermint, persimmon, pimento, pineapple, pistachio, plantain, plum, popcorn, potato, pumpkinetc, quince, rapeseed, rasberry, rice, rubber, rye, safflower, sesame, sorghum, sorghumfor, soybean, spinach, strawberry, sugarcane, sunflower, sweetpotato, tangetc, taro, tobacco, tomato, triticale, turnipfor, watermelon, wheat		mx/cierreagricola/
Montenegro	0	9	alfalfa, barley, grape, maize, oats, olive, potato, rye, wheat	2020	https://ec.europa.eu/eurostat/web/agriculture/data/database
Namibia	0	2	maize, wheat	2020	https://www.nab.com.na/agronomy/grain-statistics/
Netherlands	0	18	alfalfa, barley, broadbean, flax, grape, greencorn, hemp, maize, oats, oilseednes, potato, rapeseed, rootnes, rye, sugarbeet, sunflower, triticale, wheat	2020	https://ec.europa.eu/eurostat/web/agriculture/data/database
Pakistan	3	36	almond, apple, apricot, banana, barley, castor, chickpea, chilleetc, cotton, date, garlic, ginger, grape, groundnut, lentil, linseed, maize, mango, millet, onion, pea, peachetc, pear, plum, potato, rice, safflower, sesame, sorghum, soybean, sugarbeet, sugarcane, sunflower, tobacco, tomato, wheat	2020	https://www.mnfsr.gov.pk/Publications
Panama	12	4	bean, maize, rice, sugarcane	2020	https://www.inec.gob.pa/publicaciones/
Poland	16	25	alfalfa, barley, broadbean, flax, grape, greencorn, hemp, hop, linseed, lupin, maize, oats, oilseednes, pea, potato, pulsenes, rapeseed, rootnes, rye, soybean, sugarbeet, sunflower, tobacco, triticale, wheat	2020	https://ec.europa.eu/eurostat/web/agriculture/data/database
Portugal	0	17	barley, broadbean, grape, greencorn, lupin, maize, oats, oilseednes, olive, potato, pulsenes, rice, rootnes, rye, sunflower, triticale, wheat	2020	https://ec.europa.eu/eurostat/web/agriculture/data/database
Puerto Rico	0	67	avocado, banana, bean, cabbage, carrot, cassava, cauliflower, cherry, chestnut, chickpea, chicory, chilleetc, cinnamon, clover, cocoa, coconut, coffee, cotton, cowpea, cranberry, cucumberetc, currant, date, eggplant, fig, flax, garlic, ginger, gooseberry, grape, grapefruitetc, greencorn, greenonion, groundnut, hazelnut, hemp, lemonlime, lentil, lettuce, maize, mango, melonetc, mushroom, okra, onion, orange, papaya, pigeonpea, pimento, pineapple, plantain, pumpkinetc, quince, quinoa, rapeseed, rasberry, rice, rye, sorghum, spinach, sugarcane, sweetpotato, tangetc, taro, tomato, watermelon, yam	2020	https://www.fsa.usda.gov/news-room/efoia/electronic-reading-room/frequently-requested-information/crop-acreage-data/index
Romania	0	27	alfalfa, barley, broadbean, flax, grape, greencorn, hemp, hop, linseed, lupin, maize, oats, oilseednes, pea, potato, pulsenes, rapeseed, rice, rootnes, rye, sorghum, soybean, sugarbeet, sunflower, tobacco, triticale, wheat	2020	https://ec.europa.eu/eurostat/web/agriculture/data/database
Russia	71	6	flax, rapeseed, soybean, sugarbeet, sunflower, wheat	2020	https://rosstat.gov.ru/folder/210/document/13226
Serbia	0	19	alfalfa, barley, broadbean, grape, greencorn, maize, oats, oilseednes, potato, pulsenes, rapeseed, rootnes, rye, soybean, sugarbeet, sunflower, tobacco, triticale, wheat	2020	https://ec.europa.eu/eurostat/web/agriculture/data/database
Slovenia	0	21	alfalfa, barley, grape, greencorn, hop, maize, oats, oilseednes, olive, pea, potato, pulsenes, rapeseed, rootnes, rye, sorghum, soybean, sugarbeet, sunflower, triticale, wheat	2020	https://ec.europa.eu/eurostat/web/agriculture/data/database
South Africa	9	13	alfalfa, barley, bean, groundnut, maize, maizefor, oats, rapeseed, sorghum, soybean, sugarcane, sunflower, wheat	2017	https://www.statssa.gov.za/?page_id=1854&PPN=Report-11-02-01&SCH=7902
South Korea	14	28	apple, apricot, barley, buckwheat, cabbage,	2020	https://kosis.kr/statHtml

			chilleetc, cucumberetc, grape, groundnut, lettuce, maize, melonetc, peachetc, pear, persimmon, plum, potato, pumpkinetc, rice, sesame, soybean, spinach, strawberry, sweetpotato, tangetc, tomato, watermelon, wheat		/statHtml.do?orgId=101&tblId=DT_1EB001&vw_cd=MT_ETITLE&list_id=K1_15&scrId=&language=en&seqNo=&lang_mode=en&obj_var_id=&itm_id=&conn_path=MT_ETITLE&path=%252Feng%252FstatisticsList%252FstatisticsListIndex.do
Spain	17	27	alfalfa, barley, broadbean, grape, greencorn, hemp, hop, linseed, lupin, maize, oats, oilseednes, olive, pea, potato, pulsenes, rapeseed, rice, rootnes, rye, sorghum, soybean, sugarbeet, sunflower, tobacco, triticale, wheat	2020	https://ec.europa.eu/eurostat/web/agriculture/data/database
Switzerland	0	23	barley, broadbean, grape, greencorn, hop, linseed, lupin, maize, oats, oilseednes, pea, potato, pulsenes, rapeseed, rice, rootnes, rye, soybean, sugarbeet, sunflower, tobacco, triticale, wheat	2020	https://ec.europa.eu/eurostat/web/agriculture/data/database
Thailand	76	4	cassava, maize, pineapple, rice	2019 for rice; 2020 for other crops	http://statbbi.nso.go.th/staticreport/page/sector/en/11.aspx
U.K.	0	14	barley, broadbean, greencorn, linseed, maize, oats, oilseednes, pea, potato, rapeseed, rye, sugarbeet, triticale, wheat	2020	https://ec.europa.eu/eurostat/web/agriculture/data/database
U.S.A.	50	107	alfalfa, almond, aniseetc, apple, apricot, artichoke, asparagus, avocado, banana, barley, bean, blueberry, buckwheat, cabbage, canaryseed, carrot, cassava, cauliflower, cherry, chestnut, chickpea, chicory, chilleetc, clover, cocoa, coconut, cotton, cowpea, cranberry, cucumberetc, currant, date, eggplant, fig, flax, garlic, ginger, gooseberry, grape, grapefruitetc, greencorn, greenonion, groundnut, hazelnut, hemp, hempseed, hop, kiwi, lemonlime, lettuce, maize, maizefor, mango, melonetc, millet, mushroom, mustard, oats, okra, olive, onion, orange, papaya, peachetc, pear, peppermint, persimmon, pigeonpea, pimento, pineapple, pistachio, plantain, plum, popcorn, potato, pumpkinetc, quince, quinoa, rapeseed, rasberry, rice, rye, ryefor, safflower, sesame, sorghum, sorghumfor, sourcherry, soybean, spinach, strawberry, stringbean, sugarbeet, sugarcane, sunflower, sweetpotato, tangetc, taro, tea, tobacco, tomato, turnipfor, vetch, walnut, watermelon, wheat, yam	2020	https://www.fsa.usda.gov/news-room/efoia/electronic-reading-room/frequently-requested-information/crop-acreage-data/index
Vietnam	63	13	banana, cashew, cassava, coffee, grape, grapefruitetc, maize, mango, pepper, rice, rubber, sweetpotato, tea	2020	https://www.gso.gov.vn/en/agriculture-forestry-and-fishery/

Supplementary Table S2 Matching and aggregation of crop names for the 27 input datasets providing georeferenced crop-specific information and the FAOSTAT dataset. Names in brackets refer to the names used in the original dataset and corresponding aggregation.

Datasets	Crop names in MRF dataset
MRF [1]	abaca, agave, alfalfa, almond, aniseetc, apple, apricot, areca, artichoke, asparagus, avocado, bambara, banana, barley, bean, beetfor, berrynes, blueberry, brazil, broadbean, buckwheat, cabbage, cabbagefor, canaryseed, carob, carrot, carrotfor, cashew, cashewapple, cassava, castor, cauliflower, cerealnes, cherry, chestnut, chickpea, chicory, chilleetc, cinnamon, citrusnes, clove, clover, cocoa, coconut, coffee, cotton, cowpea, cranberry, cucumberetc, currant, date, eggplant, fibrenes, fig, flax, fonio, fornes, fruitnes, garlic, ginger, gooseberry, grape, grapefruitetc, grassnes, greenbean, greenbroadbean, greencorn, greenonion, greenpea, groundnut, hazelnut, hemp, hempseed, hop, jute, jutelikefiber, kapokfiber, kapokseed, karite, kiwi, kolanut, legumenes, lemonlime, lentil, lettuce, linseed, lupin, maize, maizefor, mango, mate, melonetc, melonseed, millet, mixedgrain, mixedgrass, mushroom, mustard, nutmeg, nutnes, oats, oilpalm, oilseedfor, oilseednes, okra, olive, onion, orange, papaya, pea, peachetc, pear, pepper, peppermint, persimmon, pigeonpea, pimento, pineapple, pistachio, plantain, plum, popcorn, poppy, potato, pulsenes, pumpkinetc, pyrethrum, quince, quinoa, ramie, rapeseed, rasberry, rice, rootnes, rubber, rye, ryefor, safflower, sesame, sisal, sorghum, sorghumfor, sourcherry, soybean, spicenes, spinach, stonefruitnes, strawberry, stringbean, sugarbeet, sugarcane, sugarnes, sunflower, swedefor, sweetpotato, tangetc, taro, tea, tobacco, tomato, triticale, tropicalnes, tung, turnipfor, vanilla, vegetablenes, vegfor, vetch, walnut, watermelon, wheat, yam, yautia
SPAM [2]	banana, barley, bean, cassava, chickpea, cocoa, coconut, coffee (arabica coffee + robusta coffee), cotton, cowpea, groundnut, lentil, maize, millet (pearl millet + small millet), oilpalm, pigeonpea, plaintain, potato, rapeseed, rice, sorghum, soybean, sugarbeet, sugarcane, sunflower, sweetpotato (sweet potato), tea, tobacco, wheat, yams
GAEZ+ 15 [3]	banana, barley, cassava, cotton, groundnut, maize, millet, oilpalm (oilpalmfruit), olive (olives), rapeseed, rice, sorghum, soybean, sugarbeet, sugarcane, sunflower, tobacco, vegfor (vegetables), wheat, yam (Yamsandotherroots)
GEOGLAM [4]	maize, rice, soybean, wheat
OILPA [5]	oilpalm (smallholder + industrial)
RAP [6]	rapeseed
EU [7]	barley, maize, oats, potato (potatoes), rapeseed (rape and turnip rape), rice, rice, soybean (soya), sugarbeet (sugar beet), sunflower, triticale, wheat (common wheat + durum wheat)
SPAMAF [8]	banana, barley, bean, cassava, chickpea, cocoa, coconut, coffee (arabica coffee + robusta coffee), cotton, cowpea, groundnut, lentil, maize, millet (pearl millet + small millet), oilpalm, pigeonpea, plaintain, potato, rapeseed, rice, sesame (sesameseed), sorghum, soybean, sugarbeet, sugarcane, sunflower, sweetpotato (sweet potato), tea, tobacco, vegfor (vegetables), wheat, yams
AFCAS [9]	cassava
SASOY [10]	soybean
MYSTHA [11]	oilpalm
ASIARICE [12]	rice
CIVGHA [13]	cocoa
UZBTJK [14]	alfalfa, barley, bean (beans), cabbage, carrot (carrots), cotton, grape (vineyard), maize, melonetc (melons), oats, onion (onions), potato (potatoes), pumpkinetc (pumpkin), rice, sorghum, soybean (soybeans), sunflower, tomato (tomatoes), vegfor (vegetables)
USA [15]	alfalfa, almond (almonds), apple (apples), apricot (apricots), asparagus, avocado (avocados), barley, bean (dry beans), blueberry (blueberries), buckwheat, cabbage, carrot (carrots + turnips), cauliflower (cauliflower + broccoli), cherry (cherries), chickpea (chick peas), chilleetc (peppers), clover (clover wildflowers), cotton, cranberry (cranberries), cucumberetc (cucumbers), eggplant (eggplants), garlic, grape (grapes), greencorn (sweet corn), groundnut (peanuts), hop (hops), lentil (lentils), lettuce, linseed (flaxseed), maize (corn), melonetc (cantaloupe + honeydew melon), millet, mustard, oats, olive (olives), onion (onions), orange (oranges), pea (peas), peachetc (nectarines + peaches), pear (pears), peppermint (mint), pistachio (pistachios), plum (plums), popcorn (pop or orn corn), potato (potatoes), pumpkinetc (gourds + pumpkins + squash), rapeseed (rape seed + canola), rice, rye, safflower, sorghum, soybean (soybeans), strawberry (strawberries), sugarbeet (sugarbeets), sugarcane, sunflower, sweetpotato (sweet potatoes), tobacco, tomato (tomatoes), triticale, vetch, walnut (walnuts), watermelon (watermelons), wheat (durum wheat + spring wheat + winter wheat + speltz)
CA [16]	barley, bean (beans), blueberry, buckwheat, canaryseed, chickpea (chick peas), cranberry, grape (vineyards), hemp, hop (hops), lentil (lentils), linseed (flaxseed), maize (corn), millet, mustard, oats, potato (potatoes), quinoa, rapeseed (canola/rapeseed), rye, safflower, sorghum, soybean (soybeans), sugarbeet (sugarbeets), sunflower, tobacco, tomato (tomatoes), triticale, vetch, wheat (wheat + winter wheat + spring wheat + spelt)
AFG [17]	cotton, rice, wheat
DEU [18]	asparagus, barley (spring barley + winter barley), carrot, grape (grapevine), hop (hops), maize

	(maize (grain) + maize), oats (spring oats), onion, potato, rapeseed (winter rapeseed), rye (winter rye), strawberry, sugarbeet (sugar beet), sunflower, wheat (winter wheat)
CHNWH [19]	wheat (winter wheat)
CHNMZ [20]	maize
CHNMZWHRI [21]	maize (single maize + rice plus maize + wheat plus maize), rice (single rice + rice plus maize + double rice x 2 + wheat plus rice), wheat (single wheat + wheat plus maize + wheat plus rice)
BGDRICE [22]	rice (Aman + Aus + Boro)
BRASUG [23]	sugarcane
SEN [24]	bambara (vouandzou), cassava, chillietc (chilli), cotton, cowpea, eggplant (aubergine), fonio, groundnut (peanut), maize, millet, okra, rice (irrigated rice + rainfed rice), sesame, sorghum, sweetpotato (sweet potato), tomato (cherry tomato + industrial tomato), watermelon
AU [25]	apple (apples), cotton, grape (grapes), rice, sugarcane, vegfor (vegetables)
FR [26]	grape (vineyards), maize (corn), rice, soybean (soy), sunflower
JP [27]	rice
CHNMZSOY [28]	maize, soybean
FAOSTAT	Abaca (manila fibre (abaca)), agave (Agave fibres nes), almond (Almonds, with shell), aniseetc (Anise, badian, fennel, coriander), apple (Apples), apricot (arpricots), areca (areca nuts), artichoke (artichokes), asparagus, avocado (avocados), Bambara (Bambara beans), banana (bananas), barley, bean (beans, dry), berrynes (berries nes), blueberry (blueberries), brazil (brazil nuts, with shell), broadbean (broad beans, horse beans, dry), buckwheat, cabbage (cabbages and other brassicas), canaryseed (canary seed), carob (carobs), carrot (carrots and turnips), cashew (cashew nuts, with shell), cashewapple, cassava, castor (castor oil seed), cauliflower (cauliflowers and broccoli), cerealnes (cereals nes), cherry (cherries), chestnut, chickpea (chick peas), chicory (chicory roots), chilleetc (chillies and peppers, green), cinnamon (cinnamon (cannella)), citrusnes (fruit, citrus nes), clove (cloves), cocoa (cocoa, beans), coconut (coconuts), coffee (coffee, green), cotton (seed cotton), cowpea (cow peas, dry), cranberry (cranberries), cucumberetc (cucumbers and gherkins), currant (currants), date (dates), eggplant (eggplants (aubergines)), fibrenes (fibre crops nes), fig (figs), flax (flax fibre and tow), fonio, fruitnes (fruit, fresh nes), garlic, ginger, gooseberry (gooseberries), grape (grapes), grapefruitetc (grapefruit (inc. pomelos)), greenbean (beans, green), greencorn (maize, green), greenonion (onions, shallots, green), greenpea (peas, green), groundnut (groundnuts, with shell), hazelnut (hazelnuts, with shell), hemp (hemp tow waste), hempseed, hop (hops), jute, kapokfiber (kapok fruit), kapokseed (kapok fruit), karite (karite nuts (sheanuts)), kiwi (kiwi fruit), kolanut (kola nuts), lemonlime (lemons and limes), lentil (lentils), lettuce (lettuce and chicory), linseed, lupin (lupins), maize, mango (mangoes, mangosteens, guavas), mate (Mat?), melonetc (melons, other (inc. cantaloupes)), melonseed, millet, mixedgrain (grain, mixed), mushroom (mushrooms and truffles), mustard (mustard seed), nutmeg (nutmeg, mace and cardamoms), nutnes (nut nes), oats, oilpalm (oil palm fruit), oilseednes (oilseeds nes), okra, olive (olives), onion (onion, dry), orange (oranges), papaya (papayas), pea (peas, dry), peachetc (peaches and nectarines), pear (pears), pepper (pepper (piper spp.)), peppermint, persimmon (persimmons), pigeonpea (pigeon peas), pineapple (pineapples), pistachio (pistachios), plantain (plaintains and others), plum (plums and sloes), poppy (poppy seed), potato (potatoes), pulsenes (pulses nes), pumpkinetc (pumpkins, squash and gourds), pyrethrum (pyrethrum, dried), quince (quinces), quinoa, ramie, rapeseed, rasberry (raspberries), rice (rice, paddy), rootnes (roots and tubers nes), rubber (rubber, natural), rye, safflower (safflower seed), sesame (sesame seed), sisal, sorghum, sourcherry (cherries, sour), soybean (soybeans), spicenes (spices nes), spinach, stonefruitnes (fruit, stone nes), strawberry (strawberries), stringbean (string beans), sugarbeet (sugar beet), sugarcane (sugar cane), sugarnes (sugar crops nes), sunflower (sunflower seed), sweetpotato (sweet potatoes), tangetc (tangerines, mandarins, clementines, satsumas), taro (taro (cocoyam)), tea, tobacco (tobacco, unmanufactured), tomato (tomatoes), triticale, tropicalnes (fruit, tropical fresh nes), tung (tung nuts), vanilla, vegfor (vegetables, fresh nes), vetch (vetches), walnut (walnuts, with shell), watermelon (watermelons), wheat, yam (yams), yautia (yautia (cocoyam))

Supplementary Table S3 The selected best-fit datasets used to construct the global maps of harvested area (*HA*) and crop area (*CA*) of each individual crop. MRF [100%] signifies that the location of the crop is not updated since 2000. Type is: T, temporary crops; and P, permanent crops. NSO refers to gap-filled data using subnational statistics from various National Statistic Offices (NSOs).

Crops	Type	Datasets selected [% contribution of the dataset to the global map]
abaca	P	MRF [100.000%]
agave	P	MRF [74.017%], NSO [25.983%]
alfalfa	T	MRF [66.358%], UZBTJK [0.010%], USA [29.587%], NSO [4.045%]
almond	P	MRF [98.685%], USA [0.772%], NSO [0.543%]
aniseetc	T	MRF [99.526%], NSO [0.474%]
apple	P	MRF [95.624%], USA [2.138%], AU [0.072%], NSO [2.166%]
apricot	P	MRF [99.321%], USA [0.031%], NSO [0.648%]
areca	P	MRF [100.000%]
artichoke	T	MRF [97.926%], NSO [2.074%]
asparagus	T	MRF [95.863%], USA [0.144%], DEU [2.765%], NSO [1.228%]
avocado	P	MRF [97.151%], USA [0.082%], NSO [2.767%]
bambara	T	MRF [95.142%], SEN [1.285%], NSO [3.573%]
banana	P	MRF [3.336%], SPAM [55.495%], GAEZ15 [21.835%], SPAMAF [19.334%]
barley	T	MRF [0.034%], SPAM [56.334%], GAEZ15 [15.588%], EU [11.843%], SPAMAF [4.747%], USA [6.474%], CA [3.324%], DEU [1.656%]
bean	T	MRF [0.001%], SPAM [82.585%], SPAMAF [14.438%], UZBTJK [0.000%], USA [2.514%], CA [0.462%]
beetfor	T	MRF [99.946%], NSO [0.054%]
berrynes	P	MRF [100.000%]
blueberry	P	MRF [73.359%], USA [23.548%], CA [1.202%], NSO [1.891%]
brazil	P	MRF [100.000%]
broadbean	T	MRF [94.638%], NSO [5.362%]
buckwheat	T	MRF [98.487%], USA [0.590%], CA [0.068%], NSO [0.856%]
cabbage	T	MRF [97.612%], USA [0.356%], NSO [2.033%]
cabbagefor	T	MRF [100.000%]
canaryseed	T	MRF [87.149%], CA [2.209%], NSO [10.642%]
carob	P	MRF [99.893%], NSO [0.107%]
carrot	T	MRF [97.009%], UZBTJK [0.000%], USA [0.426%], DEU [0.639%], NSO [1.925%]
carrotfor	T	MRF [100.000%]
cashew	P	MRF [99.789%], NSO [0.211%]
cashewapple	P	MRF [100.000%]
cassava	T	MRF [0.214%], SPAM [19.491%], GAEZ15 [45.702%], SPAMAF [23.700%], AFCAS [10.893%]
castor	T	MRF [93.774%], NSO [6.226%]
cauliflower	T	MRF [97.123%], USA [0.274%], NSO [2.603%]
cerealnes	T	MRF [100.000%]
cherry	P	MRF [97.622%], USA [1.615%], NSO [0.763%]
chestnut	P	MRF [99.907%], NSO [0.093%]
chickpea	T	MRF [4.536%], SPAM [88.978%], SPAMAF [4.461%], USA [1.693%], CA [0.300%], NSO [0.033%]
chicory	T	MRF [100.000%]
chilleetc	T	MRF [98.262%], USA [0.523%], NSO [1.215%]
cinnamon	P	MRF [100.000%]
citrusnes	P	MRF [100.000%]
clove	P	MRF [100.000%]
clover	T	MRF [85.817%], USA [13.080%], NSO [1.103%]
cocoa	P	MRF [0.187%], SPAM [88.668%], SPAMAF [8.753%], CIVGHA [2.392%]
coconut	P	MRF [0.408%], SPAM [88.469%], SPAMAF [11.123%]
coffee	P	MRF [0.128%], SPAM [99.872%]

cotton	T	MRF [1.860%], SPAM [39.252%], GAEZ15 [30.518%], SPAMAF [18.304%], USA [9.315%], AFG [0.751%]
cowpea	T	MRF [16.852%], SPAM [23.864%], SPAMAF [59.284%]
cranberry	P	MRF [90.658%], USA [3.891%], CA [0.925%], NSO [4.527%]
cucumberetc	T	MRF [98.441%], USA [0.589%], NSO [0.970%]
currant	P	MRF [100.000%]
date	P	MRF [96.623%], NSO [3.377%]
eggplant	T	MRF [98.971%], USA [0.023%], SEN [0.014%], NSO [0.992%]
fibrenes	P	MRF [100.000%]
fig	P	MRF [98.146%], NSO [1.854%]
flax	T	MRF [89.285%], NSO [10.715%]
fonio	T	MRF [98.773%], SEN [1.227%]
fornes	T	MRF [100.000%]
fruitnes	P	MRF [100.000%]
garlic	T	MRF [98.368%], USA [0.255%], NSO [1.377%]
ginger	P	MRF [100.000%], NSO [0.000%]
gooseberry	P	MRF [100.000%]
grape	P	MRF [93.637%], UZBTJK [0.002%], USA [1.735%], CA [0.088%], DEU [0.076%], AU [0.215%], FR [1.929%], NSO [2.317%]
grapefruitetc	P	MRF [99.260%], NSO [0.740%]
grassnes	T	MRF [100.000%]
greenbean	T	MRF [97.346%], NSO [2.654%]
greenbroadbean	T	MRF [99.229%], NSO [0.771%]
greencorn	T	MRF [76.401%], USA [5.330%], NSO [18.269%]
greenonion	T	MRF [96.622%], NSO [3.378%]
greenpea	T	MRF [99.182%], NSO [0.818%]
groundnut	T	MRF [0.011%], SPAM [46.145%], GAEZ15 [20.974%], SPAMAF [30.501%], USA [2.369%]
hazelnut	P	MRF [97.153%], NSO [2.847%]
hemp	T	MRF [99.477%], CA [0.321%], NSO [0.201%]
hempseed	T	MRF [100.000%]
hop	P	MRF [98.854%], USA [0.849%], CA [0.007%], DEU [0.058%], NSO [0.232%]
jute	T	MRF [93.027%], NSO [6.973%]
jutelikefiber	T	MRF [100.000%]
kapokfiber	P	MRF [100.000%]
kapokseed	P	MRF [100.000%]
karite	P	MRF [100.000%]
kiwi	P	MRF [95.682%], NSO [4.318%]
kolanut	P	MRF [100.000%]
legumenes	T	MRF [100.000%]
lemonlime	P	MRF [97.895%], NSO [2.105%]
lentil	T	MRF [0.185%], SPAM [93.185%], SPAMAF [2.031%], USA [2.100%], CA [2.498%]
lettuce	T	MRF [96.943%], USA [0.262%], NSO [2.795%]
linseed	T	MRF [91.374%], USA [1.273%], CA [0.884%], NSO [6.469%]
lupin	T	MRF [86.430%], NSO [13.570%]
maize	T	MRF [0.000%], SPAM [9.016%], GAEZ15 [40.796%], GEOGLAM [11.392%], EU [6.451%], SPAMAF [14.573%], USA [11.327%], CA [0.873%], CHNMZ [5.571%]
maizefor	T	MRF [90.049%], NSO [9.951%]
mango	P	MRF [97.440%], NSO [2.560%]
mate	P	MRF [100.000%]
melonetc	T	MRF [96.573%], UZBTJK [0.001%], USA [0.335%], NSO [3.091%]
melonseed	T	MRF [100.000%]
millet	T	MRF [0.068%], SPAM [62.049%], GAEZ15 [12.150%], SPAMAF [25.695%], CA [0.039%]
mixedgrain	T	MRF [94.313%], NSO [5.687%]
mixedgrass	T	MRF [100.000%]

mushroom	T	MRF [97.790%], NSO [2.210%]
mustard	T	MRF [98.991%], USA [0.657%], CA [0.352%]
nutmeg	P	MRF [100.000%]
nutnes	P	MRF [100.000%]
oats	T	MRF [70.492%], EU [9.372%], UZBTJK [0.000%], USA [11.864%], CA [2.463%], DEU [1.480%], NSO [4.330%]
oilpalm	P	MRF [1.631%], SPAM [6.712%], GAEZ15 [7.148%], OIPA [39.624%], SPAMAF [44.886%]
oilseedfor	T	MRF [100.000%]
oilseednes	T	MRF [98.928%], NSO [1.072%]
okra	T	MRF [91.037%], SEN [0.436%], NSO [8.527%]
olive	P	MRF [20.507%], GAEZ15 [76.605%], USA [2.142%], NSO [0.745%]
onion	T	MRF [96.549%], UZBTJK [0.000%], USA [0.551%], DEU [0.478%], NSO [2.421%]
orange	P	MRF [98.157%], USA [0.337%], NSO [1.506%]
papaya	P	MRF [98.685%], NSO [1.315%]
pea	T	MRF [89.569%], USA [3.041%], CA [1.833%], NSO [5.557%]
peachetc	P	MRF [96.579%], USA [1.467%], NSO [1.954%]
pear	P	MRF [98.553%], USA [0.368%], NSO [1.078%]
pepper	P	MRF [100.000%]
peppermint	T	MRF [51.551%], USA [48.449%]
persimmon	P	MRF [95.232%], NSO [4.768%]
pigeonpea	T	MRF [0.131%], SPAM [95.843%], SPAMAF [4.026%]
pimento	P	MRF [99.553%], NSO [0.447%]
pineapple	P	MRF [99.283%], NSO [0.717%]
pistachio	P	MRF [98.763%], USA [1.133%], NSO [0.104%]
plantain	P	MRF [0.018%], SPAM [59.807%], SPAMAF [40.175%]
plum	P	MRF [98.444%], USA [0.359%], NSO [1.197%]
popcorn	T	USA [100.000%]
poppy	T	MRF [100.000%]
potato	T	MRF [0.352%], SPAM [78.839%], EU [5.648%], SPAMAF [11.601%], USA [2.305%], CA [0.279%], DEU [0.977%]
pulsenes	T	MRF [99.618%], NSO [0.382%]
pumpkinetc	T	MRF [94.839%], UZBTJK [0.000%], USA [1.540%], NSO [3.622%]
pyrethrum	T	MRF [100.000%]
quince	P	MRF [98.897%], NSO [1.103%]
quinoa	T	MRF [97.206%], CA [0.130%], NSO [2.665%]
ramie	P	MRF [100.000%]
rapeseed	T	MRF [0.020%], SPAM [55.132%], GAEZ15 [23.482%], RAP [3.737%], EU [11.837%], SPAMAF [1.624%], USA [2.557%], DEU [1.603%], NSO [0.008%]
rasberry	P	MRF [92.855%], NSO [7.145%]
rice	T	MRF [0.024%], SPAM [18.434%], GAEZ15 [23.037%], GEOGLAM [17.406%], EU [0.177%], SPAMAF [24.242%], ASIARICE [0.975%], USA [1.597%], CHNMZWHRI [12.141%], BGDRICE [0.506%], FR [0.015%], JP [1.445%]
rootnes	T	MRF [99.948%], NSO [0.052%]
rubber	P	MRF [99.761%], NSO [0.239%]
rye	T	MRF [79.607%], EU [9.392%], USA [8.833%], CA [0.489%], DEU [1.382%], NSO [0.297%]
ryefor	T	MRF [65.982%], NSO [34.018%]
safflower	T	MRF [95.419%], USA [1.376%], NSO [3.205%]
sesame	T	MRF [92.309%], SEN [0.060%], NSO [7.630%]
sisal	P	MRF [100.000%]
sorghum	T	MRF [0.011%], SPAM [62.104%], GAEZ15 [15.631%], SPAMAF [22.180%], UZBTJK [0.001%], CA [0.030%], NSO [0.043%]
sorghumfor	T	MRF [75.275%], NSO [24.725%]
sourcherry	P	MRF [93.743%], NSO [6.257%]
soybean	T	MRF [0.004%], SPAM [7.436%], GAEZ15 [19.498%], GEOGLAM [8.338%], EU [3.887%], SPAMAF [14.948%], SASOY [9.584%], UZBTJK [0.000%], USA [14.031%], CA [1.146%], FR [0.797%], CHNMZSOY [20.332%]

spicenes	P	MRF [100.000%]
spinach	T	MRF [95.198%], NSO [4.802%]
stonefruitnes	P	MRF [100.000%]
strawberry	P	MRF [94.463%], USA [0.422%], DEU [2.012%], NSO [3.103%]
stringbean	T	MRF [99.758%], NSO [0.242%]
sugarbeet	T	MRF [0.431%], SPAM [54.143%], GAEZ15 [24.381%], EU [19.117%], SPAMAF [1.770%], CA [0.159%]
sugarcane	T	MRF [10.673%], SPAM [28.840%], GAEZ15 [35.040%], SPAMAF [14.886%], USA [0.928%], BRASUG [9.604%], NSO [0.028%]
sugarnes	T	MRF [100.000%]
sunflower	T	MRF [0.050%], SPAM [55.824%], GAEZ15 [14.310%], EU [15.797%], SPAMAF [8.464%], USA [3.246%], CA [0.131%], FR [2.176%]
swedefor	T	MRF [100.000%]
sweetpotato	T	MRF [2.558%], SPAM [67.858%], SPAMAF [29.004%], USA [0.581%]
tangetc	P	MRF [98.323%], NSO [1.677%]
taro	T	MRF [98.088%], NSO [1.912%]
tea	P	MRF [8.303%], SPAM [79.299%], SPAMAF [12.398%]
tobacco	T	MRF [0.048%], SPAM [34.140%], GAEZ15 [41.044%], SPAMAF [23.146%], USA [1.598%], CA [0.024%]
tomato	T	MRF [97.880%], UZBTJK [0.000%], USA [0.354%], CA [0.048%], SEN [0.001%], NSO [1.717%]
triticale	T	MRF [71.471%], EU [8.907%], USA [18.367%], CA [0.441%], NSO [0.814%]
tropicalnes	P	MRF [100.000%]
tung	P	MRF [100.000%]
turnipfor	T	MRF [85.889%], NSO [14.111%]
vanilla	P	MRF [100.000%]
vegetablenes	T	MRF [100.000%]
vegfor	T	MRF [0.007%], GAEZ15 [86.098%], SPAMAF [13.650%], UZBTJK [0.002%], AU [0.244%]
vetch	T	MRF [99.765%], USA [0.148%], CA [0.052%], NSO [0.035%]
walnut	P	MRF [99.149%], USA [0.503%], NSO [0.348%]
watermelon	T	MRF [97.830%], USA [0.597%], SEN [0.309%], NSO [1.263%]
wheat	T	MRF [0.561%], SPAM [27.370%], GAEZ15 [11.204%], GEOGLAM [21.840%], EU [7.991%], SPAMAF [6.137%], USA [14.899%], CA [3.103%], DEU [1.288%], CHNWH [5.605%]
yam	T	MRF [0.019%], SPAM [49.334%], GAEZ15 [30.448%], SPAMAF [20.198%]
yautia	T	MRF [99.177%], NSO [0.823%]

Supplementary Table S4: Endogenous dataset quality indicators of national statistical offices (NSO) datasets described in Supplementary Table S1.

Country/ Territory name	Synchrony Q_y	Administration Q_a	Source Q_s	Validation Q_v	Resolution Q_r	Maturity Q_m	Dispatch Q_d	$\sum_k Q_k$
Albania	1	1	0.5	0	0	0	1	3.5
Algeria	0.75	1	0.5	0	0	0	1	3.25
Argentina	1	1	0.5	0	0	0	1	3.5
Armenia	1	1	0.5	0	0	0	1	3.5
Australia	1	1	0.5	0	0	0	1	3.5
Austria	1	1	0.5	0	0	0	1	3.5
Azerbaijan	1	1	0.5	0	0	0	1	3.5
Bangladesh	1	1	0.5	0	0	0	1	3.5
Belarus	1	1	0.5	0	0	0	1	3.5
Belgium	1	1	0.5	0	0	0	1	3.5
Bosnia and Herzegovina	1	1	0.5	0	0	0	1	3.5
Botswana	0.95	1	0.5	0	0	0	1	3.45
Brunei	1	1	0.5	0	0	0	1	3.5
Bulgaria	1	1	0.5	0	0	0	1	3.5
Burkina Faso	1	1	0.5	0	0	0	1	3.5
Cambodia	0.95	1	0.5	0	0	0	1	3.45
Cameroon	0.95	1	0.5	0	0	0	1	3.45
Canada	1	1	0.5	0	0	0	1	3.5
Chile	1	1	0.5	0	0	0	1	3.5
China	1	1	0.5	0	0	0	1	3.5
Colombia	1	1	0.5	0	0	0	1	3.5
Costa Rica	1	1	0.5	0	0	0	1	3.5
Croatia	1	1	0.5	0	0	0	1	3.5
Cyprus	1	1	0.5	0	0	0	1	3.5
Czech Republic	1	1	0.5	0	0	0	1	3.5
D.R. Congo	0.9	1	0.5	0	0	0	1	3.4
Denmark	1	1	0.5	0	0	0	1	3.5
Dominican Republic	1	1	0.5	0	0	0	1	3.5
Ecuador	1	1	0.5	0	0	0	1	3.5
Egypt	0.85	1	0.5	0	0	0	1	3.35
Estonia	1	1	0.5	0	0	0	1	3.5
FYR Macedonia	1	1	0.5	0	0	0	1	3.5
Finland	1	1	0.5	0	0	0	1	3.5
France	1	1	0.5	0	0	0	1	3.5
Germany	1	1	0.5	0	0	0	1	3.5
Greece	1	1	0.5	0	0	0	1	3.5
Guam	1	1	0.5	0	0	0	1	3.5
Hungary	1	1	0.5	0	0	0	1	3.5
Iceland	1	1	0.5	0	0	0	1	3.5
India	1	1	0.5	0	0	0	1	3.5
Iran	0.7	1	0.5	0	0	0	1	3.2
Ireland	1	1	0.5	0	0	0	1	3.5
Italy	1	1	0.5	0	0	0	1	3.5
Japan	1	1	0.5	0	0	0	1	3.5
Laos	1	1	0.5	0	0	0	1	3.5
Latvia	1	1	0.5	0	0	0	1	3.5
Lithuania	1	1	0.5	0	0	0	1	3.5
Luxembourg	1	1	0.5	0	0	0	1	3.5
Malaysia	1	1	0.5	0	0	0	1	3.5
Malta	1	1	0.5	0	0	0	1	3.5
Mexico	0.9	1	0.5	0	0	0	1	3.4
Montenegro	1	1	0.5	0	0	0	1	3.5
Namibia	1	1	0.5	0	0	0	1	3.5
Netherlands	1	1	0.5	0	0	0	1	3.5
Pakistan	1	1	0.5	0	0	0	1	3.5
Panama	1	1	0.5	0	0	0	1	3.5
Poland	1	1	0.5	0	0	0	1	3.5
Portugal	1	1	0.5	0	0	0	1	3.5
Puerto Rico	1	1	0.5	0	0	0	1	3.5
Romania	1	1	0.5	0	0	0	1	3.5
Russia	1	1	0.5	0	0	0	1	3.5

Serbia	1	1	0.5	0	0	0	1	3.5
Slovenia	1	1	0.5	0	0	0	1	3.5
South Africa	0.85	1	0.5	0	0	0	1	3.35
South Korea	1	1	0.5	0	0	0	1	3.5
Spain	1	1	0.5	0	0	0	1	3.5
Switzerland	1	1	0.5	0	0	0	1	3.5
Thailand	1	1	0.5	0	0	0	1	3.5
U.K.	1	1	0.5	0	0	0	1	3.5
U.S.A.	1	1	0.5	0	0	0	1	3.5
Vietnam	1	1	0.5	0	0	0	1	3.5

Supplementary Table S5: Summary of dataset validation.

	Acronym	Validation description	Q_v description	Reference
1	MRF	Evaluation based on previous crop maps from USDA and Leff et al. (2004).	Intermediate	Monfreda et al. (2008) [1]
2	SPAM	Partially evaluated against ground-truth data.	Intermediate	Yu et al. (2020) [2]
3	GAEZ+2015	Evaluated against FAOSTAT and Global Dataset of Historical Yields.	Intermediate	Grogan et al. (2022) [3]
4	GEOGLAM	Evaluated against sub-national official statistics.	Intermediate	Becker-Reshef et al. (2022) [4]
5	OIPA	Evaluated against ground-truth data.	High	Descals et al. (2021) [5]
6	RAP	Evaluated against previous rapeseed geospatial products.	Intermediate	Han, et al. (2021) [6]
7	EU	Evaluated against LUCAS core points, annual crop declarations by EU farmers, and official subnational statistics.	High	d'Andrimont et al. (2021) [7]
8	SPAMAF	N/A	None	IFPRI (2020) [8]
9	AFCAS	N/A	None	Szyniszewska (2020) [9]
10	SASOY	Evaluated against ground-truth data.	High	Song et al. (2021) [10]
11	MYSTHA	Evaluated against very high-resolution satellite imagery using visual interpretation.	Intermediate	Danylo et al. (2021) [11]
12	ASIARICE	Evaluated against FAOSTAT and official subnational statistics.	Intermediate	Han et al. (2022) [12]
13	CIVGHA	Evaluated against ground-truth data.	High	Abu et al. (2021) [13]
14	UZBTJK	Evaluated against ground-truth data.	High	Remelgado et al. (2020) [14]
15	USA	Evaluated against ground-truth data.	High	Boryan et al. (2011) [15]
16	CA	Evaluated against ground-truth data.	High	Fisette et al. (2013) [16]
17	AFG	Evaluated against ground-truth data.	High	FAO (2021) [17]
18	DEU	Visually quality checked against Google Earth high resolution imagery.	Intermediate	Blickensdörfer et al. (2022) [18]
19	CHNWH	Evaluated against ground-truth data.	High	Dong et al. (2020) [19]
20	CHNMZ	Evaluated against ground-truth data.	High	Qiu et al. (2018) [20]
21	CHNMZWHRI	Evaluated against ground-truth data.	High	Qiu et al. (2022) [21]
22	BGDRICE	Evaluated against ground-truth data.	High	Singha et al. (2019) [22]
23	BRA	Evaluated against high-resolution images from Google Earth.	Intermediate	Zheng et al. (2022) [23]
24	SEN	Evaluated against ground-truth data.	High	FAO (2021) [24]
25	AU	N/A	None	ABARES (2022) [25]
26	FR	Evaluated against ground-truth data.	High	Thierion et al. (2022) [26]
27	JP	Evaluated against ground-truth data.	High	JAXA EORC (2021) [27]
28	CHNMZSOY	Evaluated against ground-truth data.	High	Li et al. (2023) [28]

Supplementary Figures

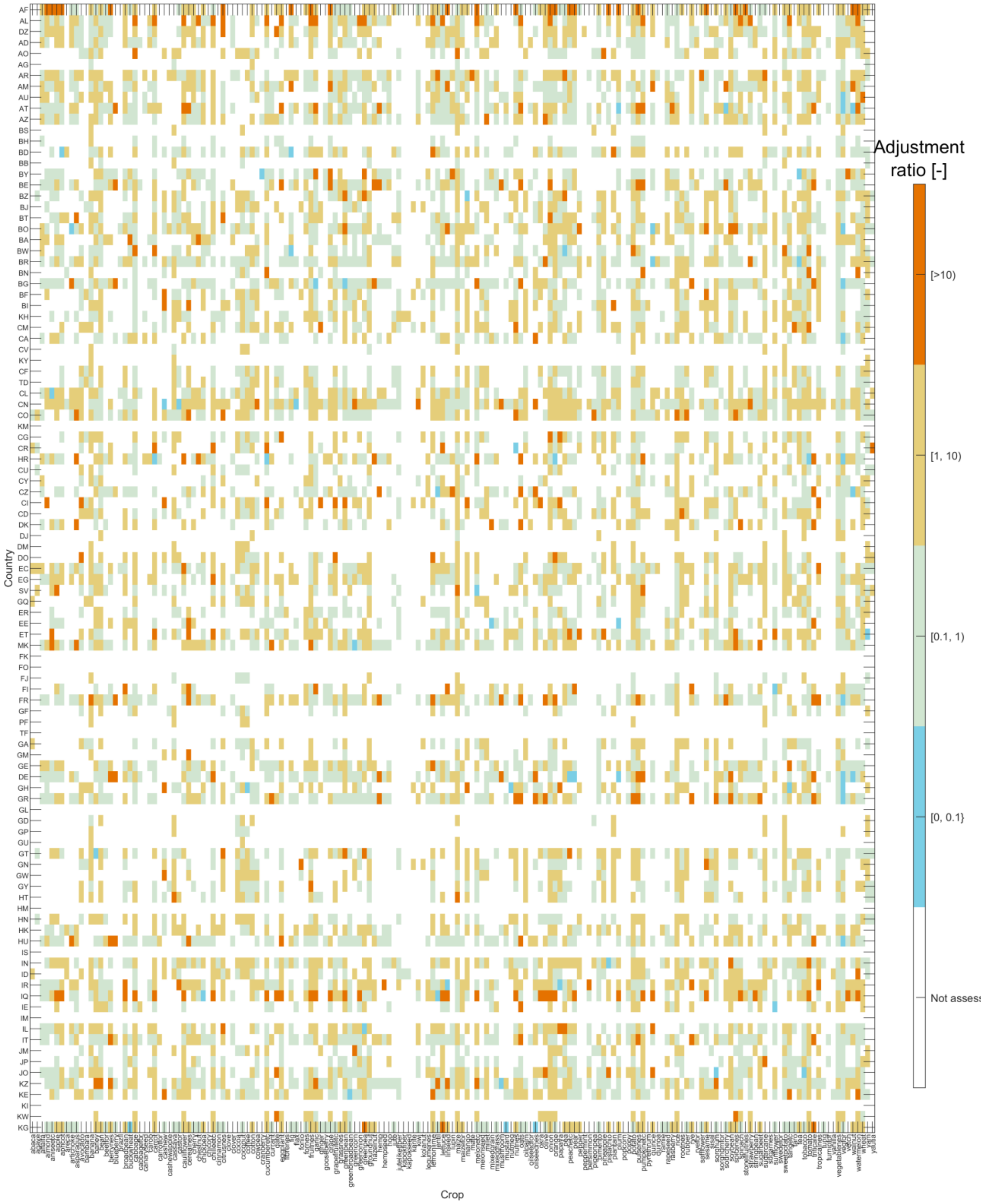

Supplementary Figure S1. The "after-to-before" ratio used in Step 6 to adjust harvested area for matching national-level FAOSTAT data for 2020.

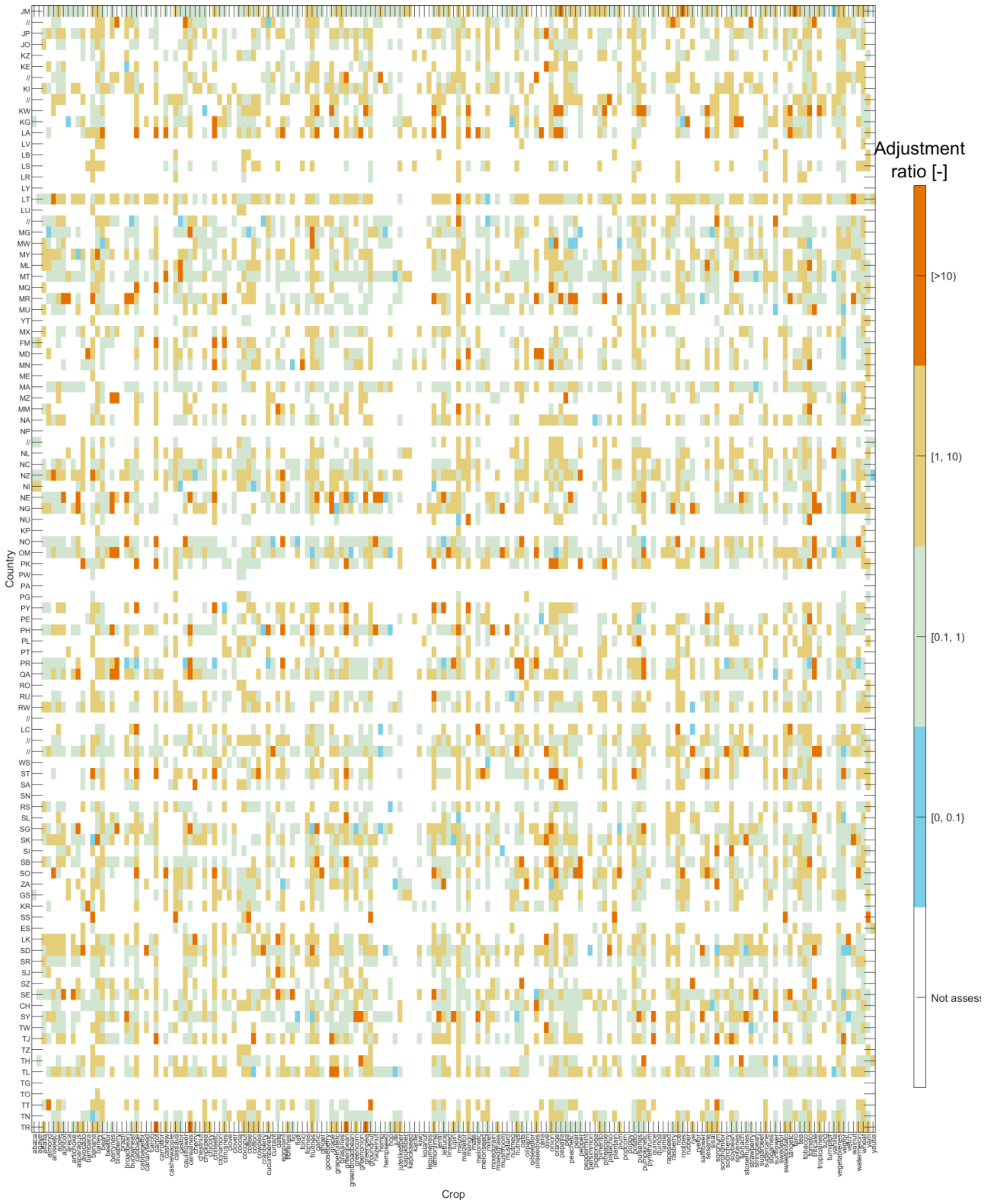

Continued Supplementary Figure S1. The "after-to-before" ratio used in Step 6 to adjust harvested area for matching national-level FAOSTAT data for 2020.

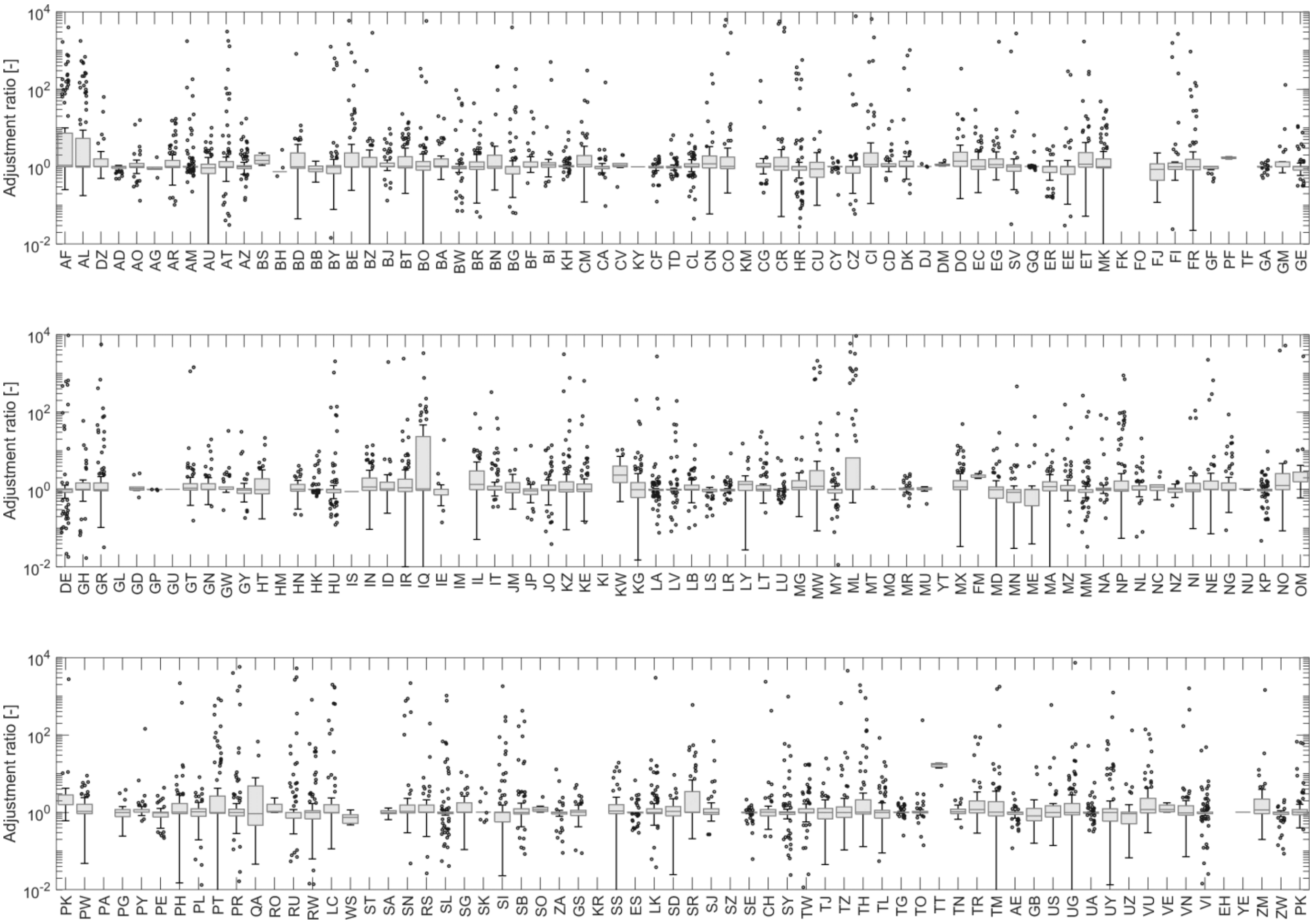

Supplementary Figure S2. Representation of full statistics of the "after-to-before" ratio used in Step 6 to adjust harvested area for matching national-level FAOSTAT data for 2020 by countries.

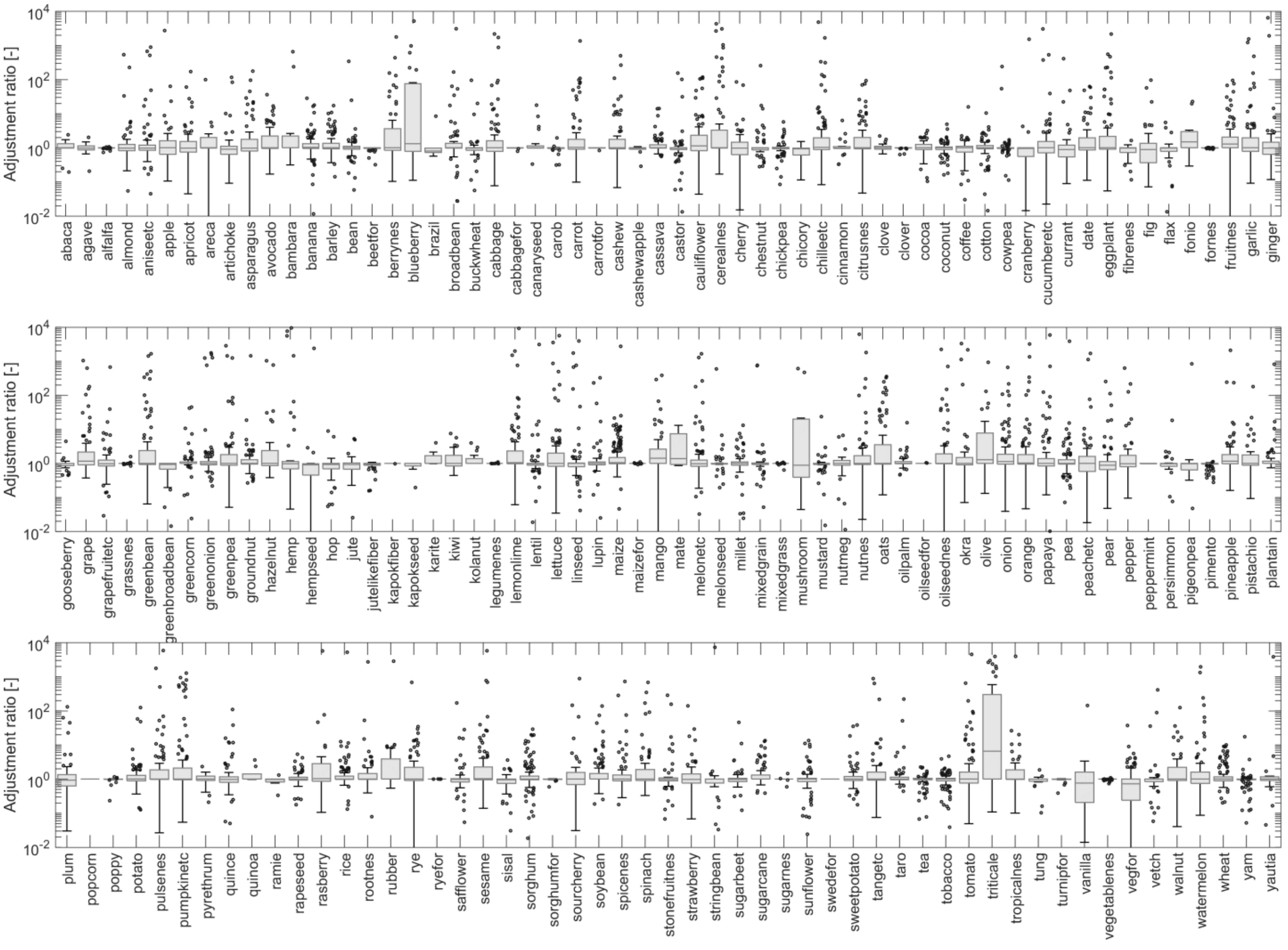

Supplementary Figure S3. Representation of full statistics of the “after-to-before” ratio used in Step 6 to adjust harvested area for matching national-level FAOSTAT data for 2020 by crops.

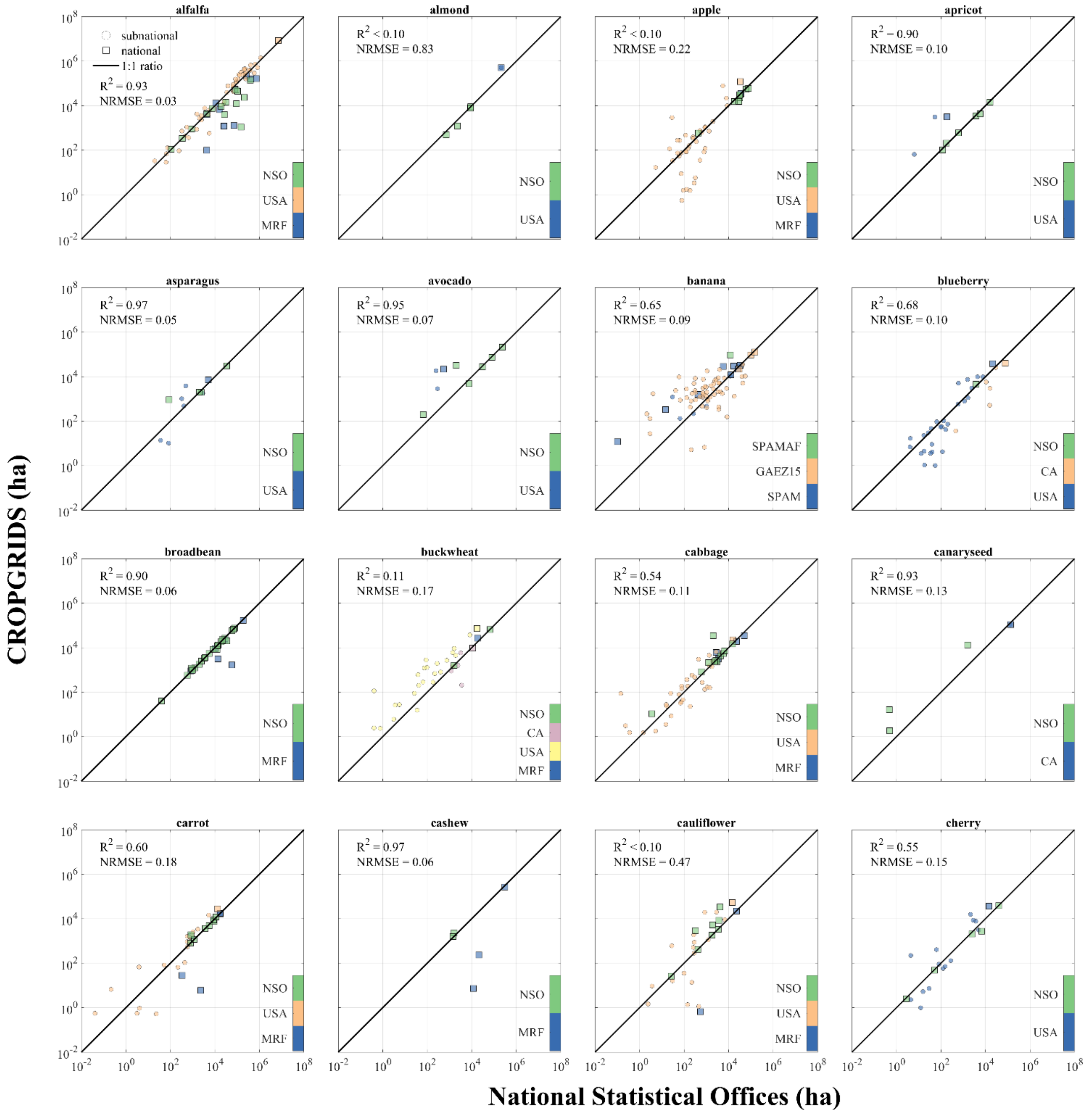

Supplementary Figure S4. Validation of crop harvested areas at national and subnational levels between CROPGRIDS and National Statistical Offices. The colours of the markers refer to the georeferenced datasets selected to use in CROPGRIDS.

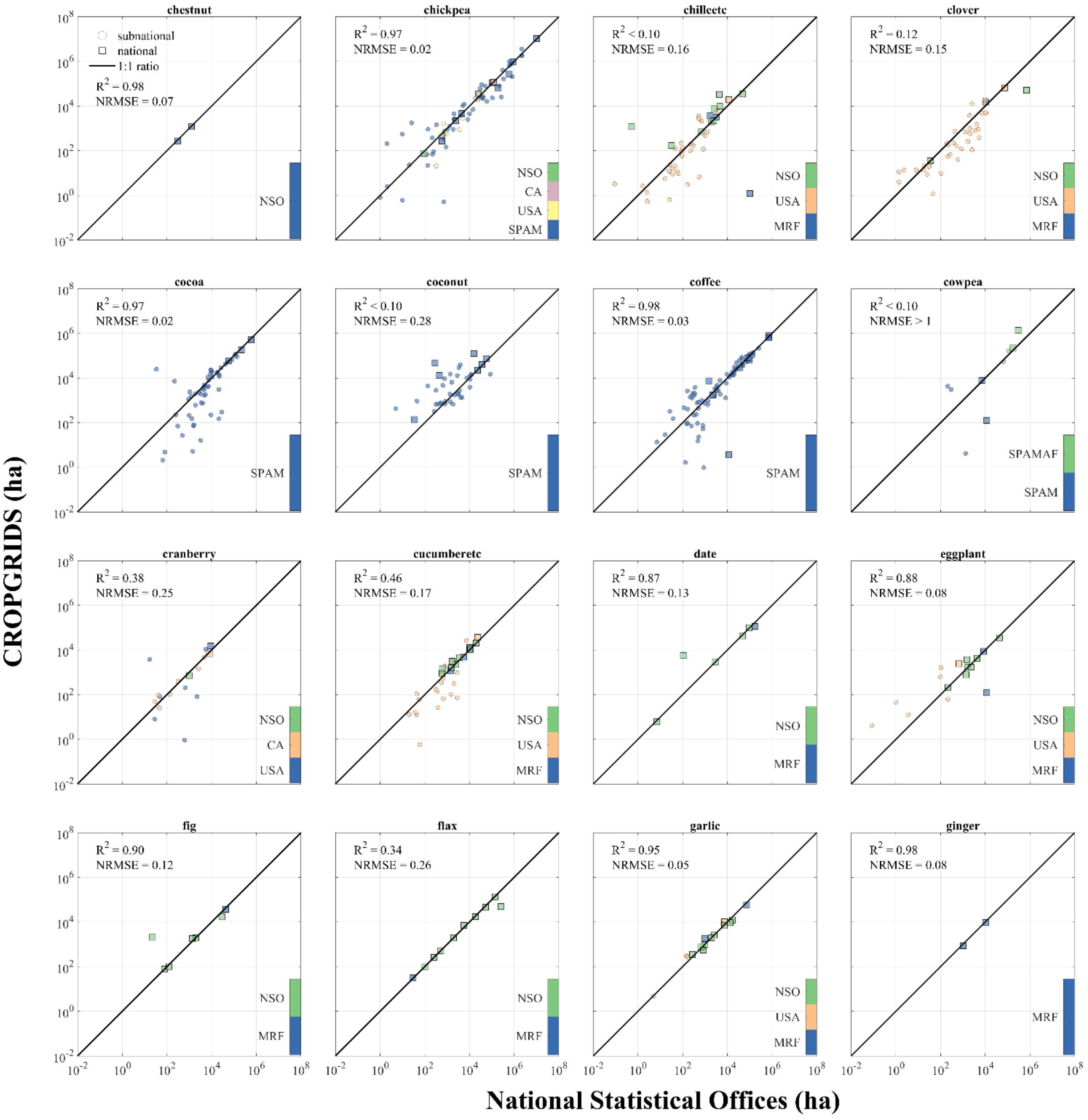

continued Supplementary Figure S4. Validation of crop harvested areas at national and subnational levels between CROPGRIDS and National Statistical Offices. The colours of the markers refer to the georeferenced datasets selected to use in CROPGRIDS.

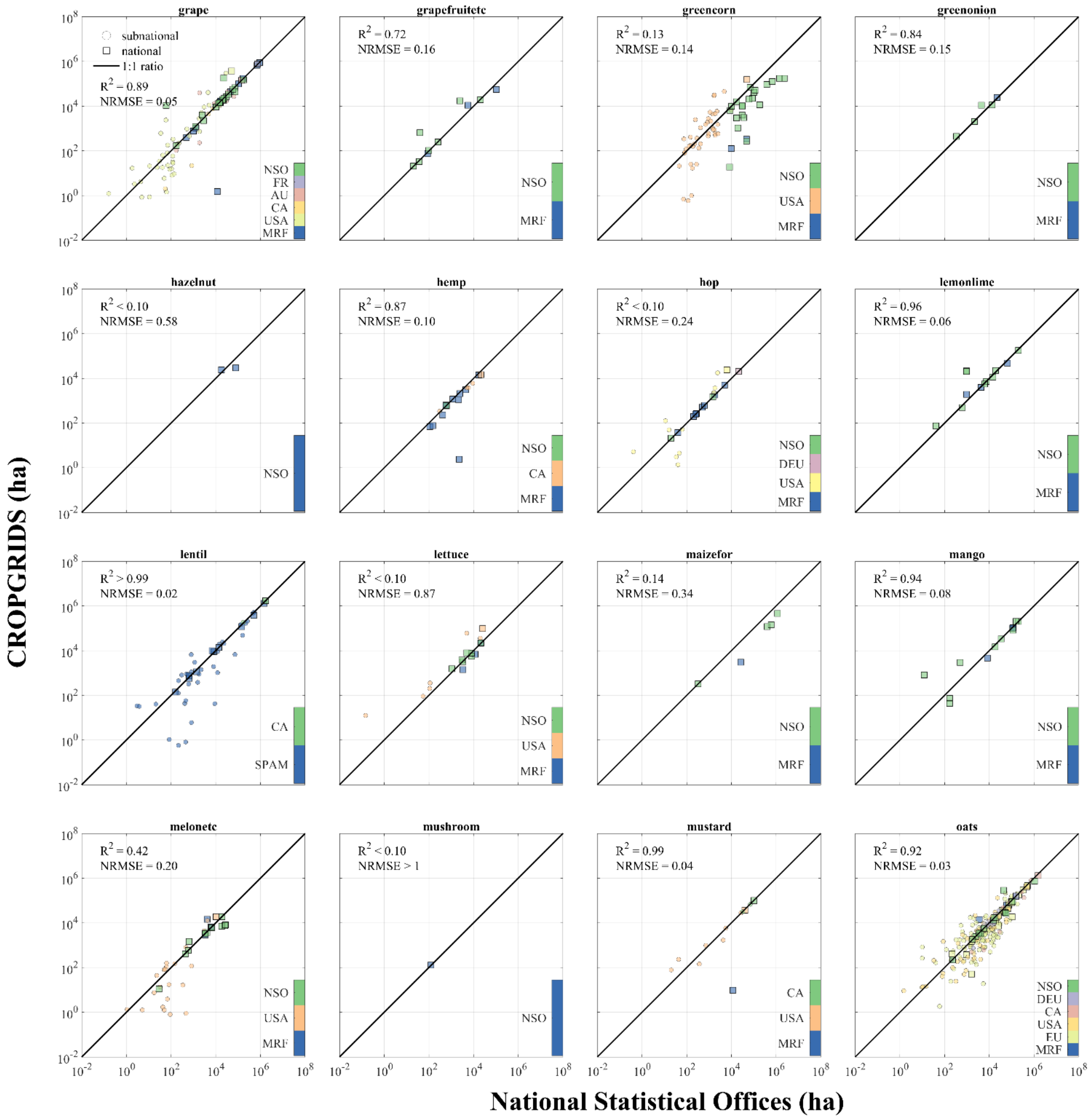

continued Supplementary Figure S4. Validation of crop harvested areas at national and subnational levels between CROPGRIDS and National Statistical Offices. The colours of the markers refer to the georeferenced datasets selected to use in CROPGRIDS.

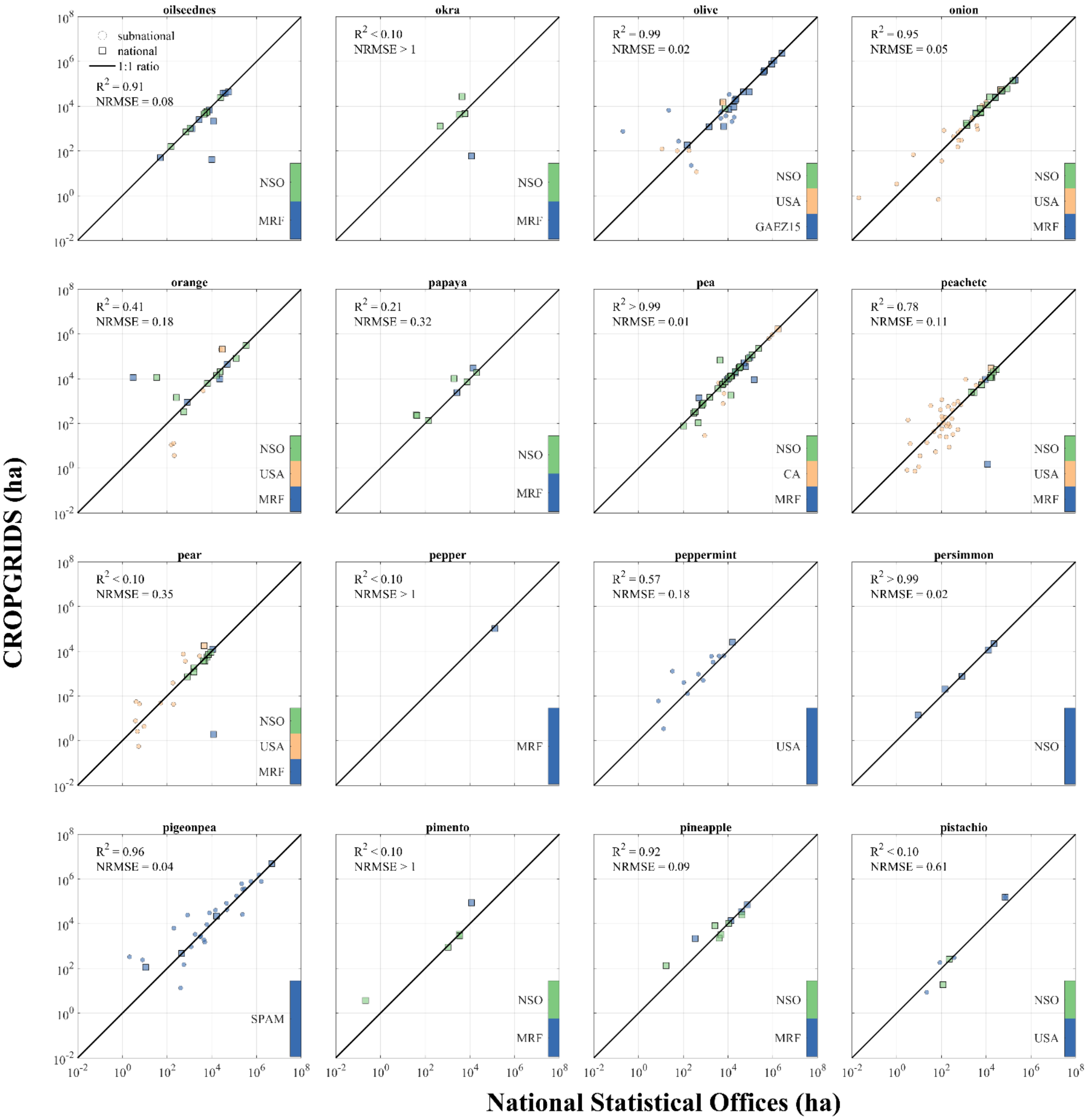

continued Supplementary Figure S4. Validation of crop harvested areas at national and subnational levels between CROPGRIDS and National Statistical Offices. The colours of the markers refer to the georeferenced datasets selected to use in CROPGRIDS.

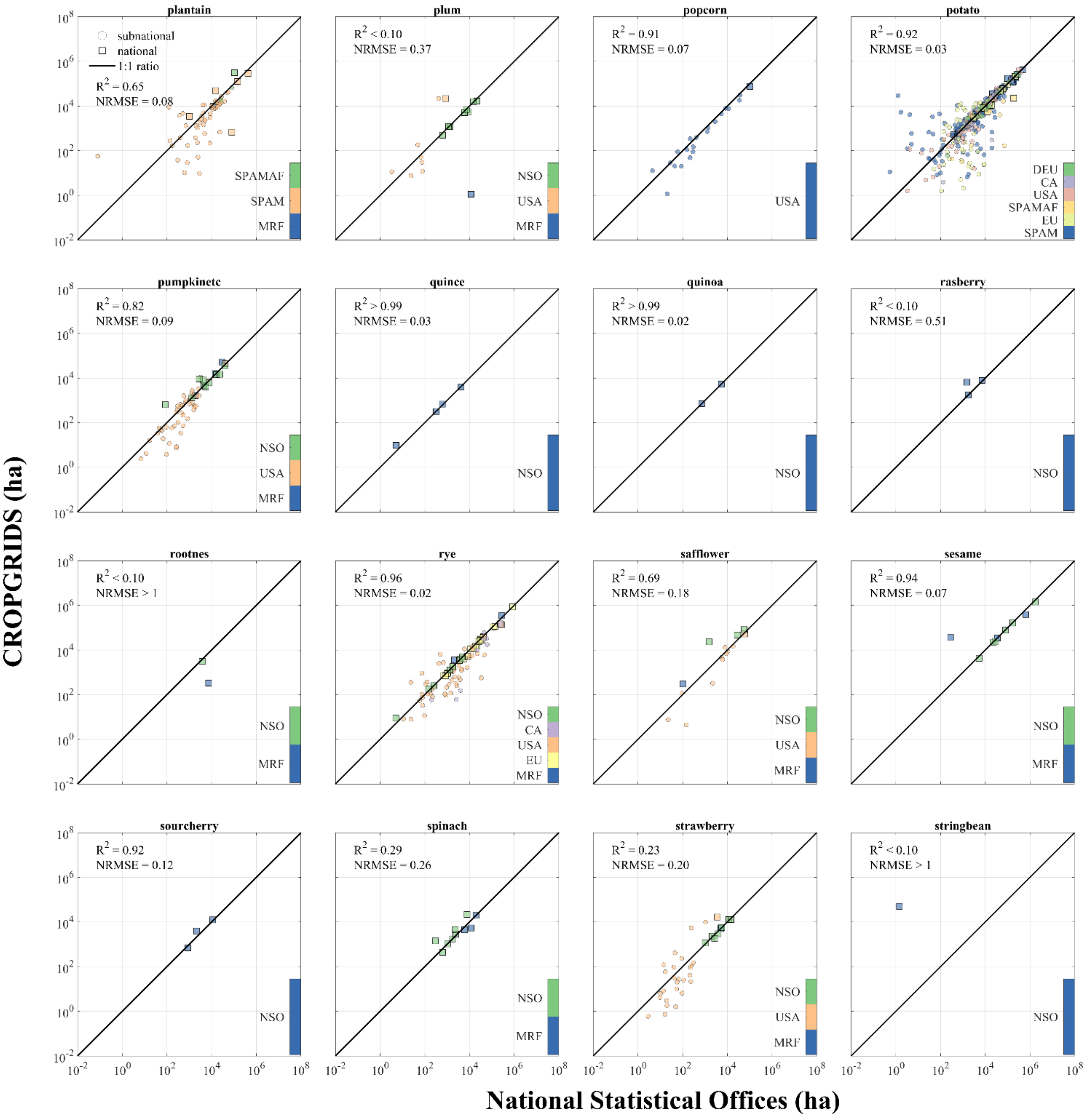

continued Supplementary Figure S4. Validation of crop harvested areas at national and subnational levels between CROPGRIDS and National Statistical Offices. The colours of the markers refer to the georeferenced datasets selected to use in CROPGRIDS.

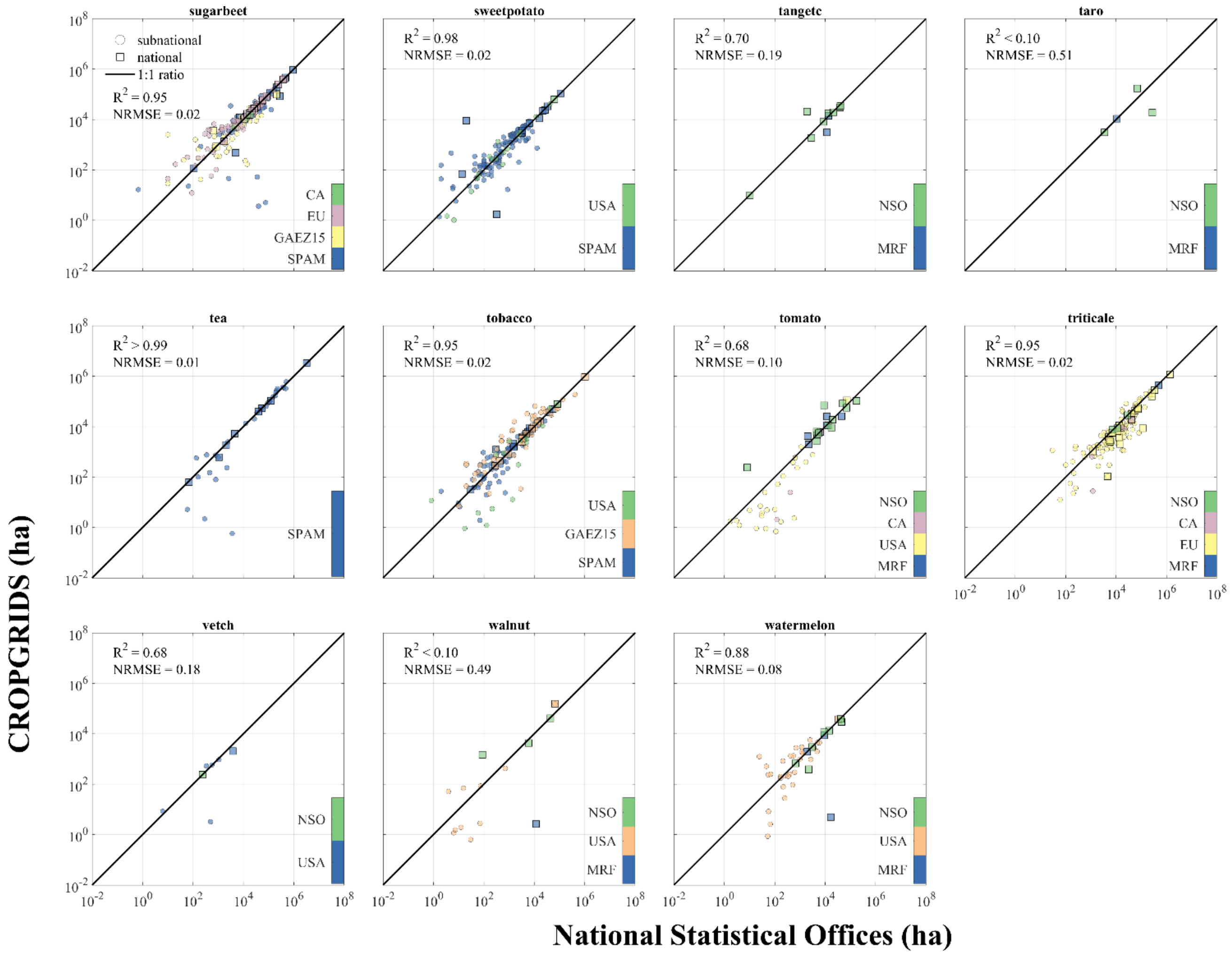

continued Supplementary Figure S4. Validation of crop harvested areas at national and subnational levels between CROPGRIDS and National Statistical Offices. The colours of the markers refer to the georeferenced datasets selected to use in CROPGRIDS.

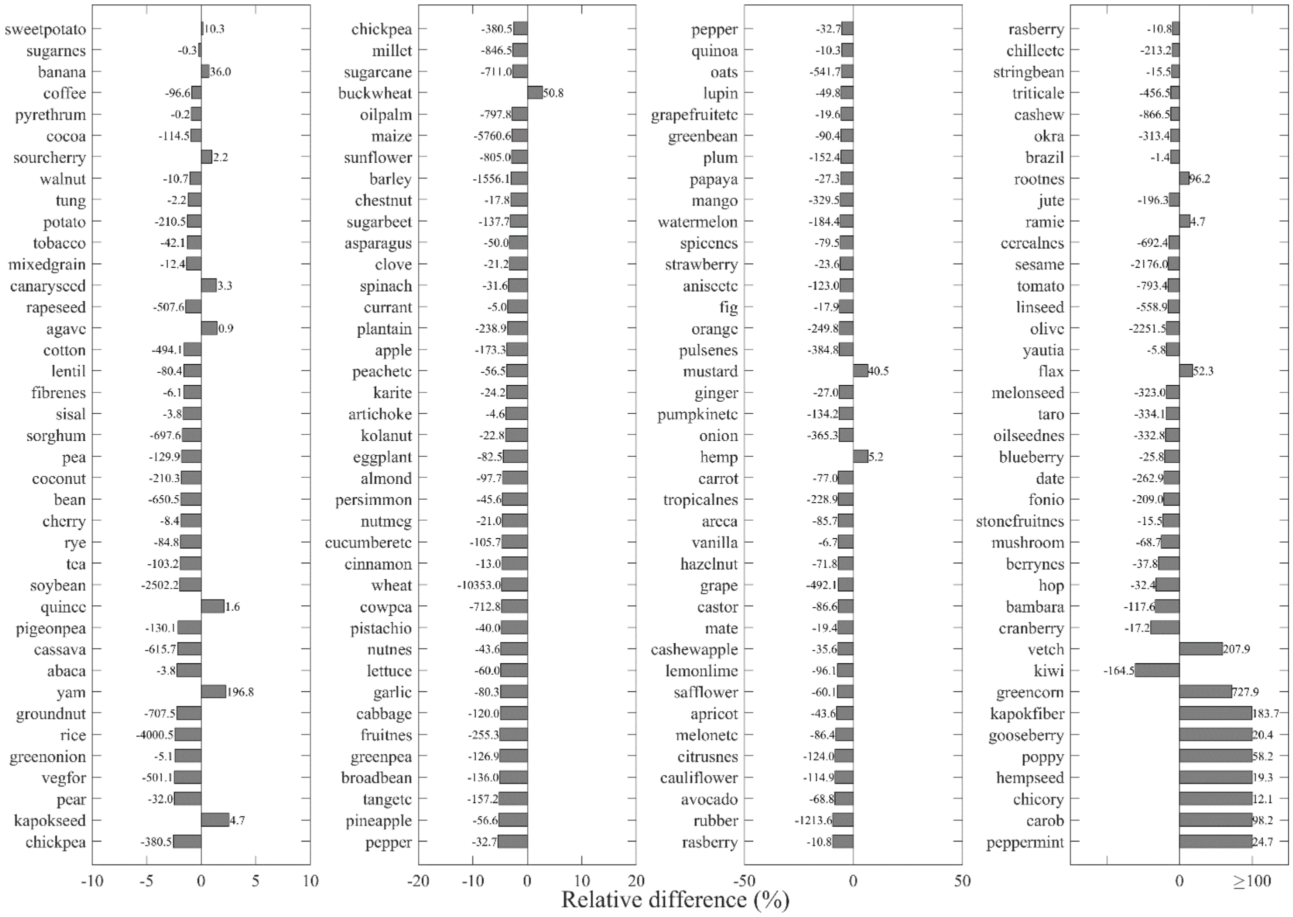

Supplementary Figure S5. Relative differences of global harvested area between CROPGRIDS and FAOSTAT for 153 crops. Positive and negative bars and numbers signify that CROPGRIDS either overestimated or underestimated harvested area compared to FAOSTAT. The numbers next to the bars represent differences [1000×ha].

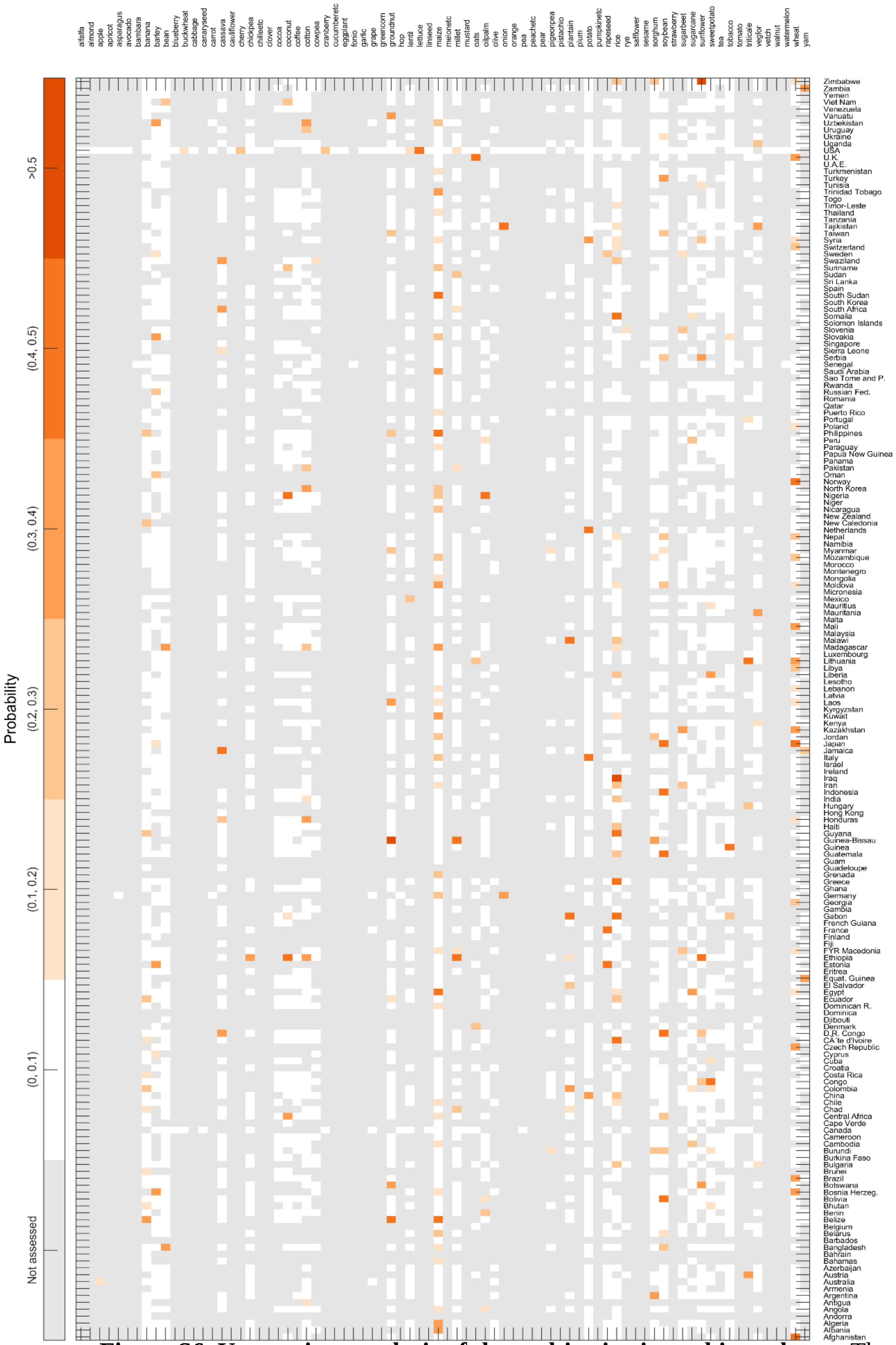

Supplementary Figure S6. Uncertainty analysis of the multi-criteria ranking scheme. The mosaic represents the probability that randomizing the weights of the endogenous and exogenous characteristics returns the selection of a different best-fit dataset in countries and crops where more than one dataset is available.

References

[1] Monfreda, C., Ramankutty, N. & Foley, J. A. Farming the planet: 2. Geographic distribution of crop areas, yields, physiological types, and net primary production in the year 2000. Global Biogeochem Cycles 22, GB1022 (2008). https://doi.org/10.1029/2007gb002947

[2] Yu, Q. Y. et al. A cultivated planet in 2010-Part 2: The global gridded agricultural-production maps. Earth Syst Sci Data 12, 3545-3572 (2020). https://doi.org/10.5194/essd-12-3545-2020

[3] Grogan, D., Frolking, S., Wisser, D., Prusevich, A. & Glidden, S. Global gridded crop harvested area, production, yield, and monthly physical area data circa 2015. Sci Data 9, 15 (2022). https://doi.org/10.1038/s41597-021-01115-2

[4] Becker-Reshef, I. et al. GEOGLAM Best Available Crop Type Masks. Zenodo https://doi.org/10.5281/ZENODO.6511594 (2022).

[5] Descals, A. et al. High-resolution global map of smallholder and industrial closed-canopy oil palm plantations. Earth Syst Sci Data 13, 1211-1231 (2021). https://doi.org/10.5194/essd-13-1211-2021

[6] Han, J. C. et al. The RapeseedMap10 database: annual maps of rapeseed at a spatial resolution of 10m based on multi-source data. Earth Syst Sci Data 13, 2857-2874 (2021). https://doi.org/10.5194/essd-13-2857-2021

[7] d'Andrimont, R. et al. From parcel to continental scale-A first European crop type map based on Sentinel-1 and LUCAS Copernicus in-situ observations. Remote Sens Environ 266, 112708 (2021). https://doi.org/10.1016/j.rse.2021.112708

[8] IFPRI. Spatially-disaggregated crop production statistics data in Africa South of the Sahara for 2017 (Version 3.0). Harvard Dataverse https://doi.org/10.7910/DVN/FSSKBW (2020)

[9] Szyniszewska, A. M. CassavaMap, a fine-resolution disaggregation of cassava production and harvested area in Africa in 2014. Sci Data 7, 159 (2020). https://doi.org/10.1038/s41597-020-0501-z

[10] Song, X. P. et al. Massive soybean expansion in South America since 2000 and implications for conservation. Nat Sustain 4, 784-792 (2021). https://doi.org/10.1038/s41893-021-00729-z

[11] Danylo, O. et al. A map of the extent and year of detection of oil palm plantations in Indonesia, Malaysia and Thailand. Sci Data 8, 96 (2021). https://doi.org/10.1038/s41597-021-00867-1

[12] Han, J. C. et al. Annual paddy rice planting area and cropping intensity datasets and their dynamics in the Asian monsoon region from 2000 to 2020. Agr Syst 200, 103437 (2022). https://doi.org/10.1016/j.agsy.2022.103437

[13] Abu, I. O., Szantoi, Z., Brink, A., Robuchon, M. & Thiel, M. Detecting cocoa plantations in Cote d'Ivoire and Ghana and their implications on protected areas. Ecol Indic 129, 107863 (2021). https://doi.org/10.1016/j.ecolind.2021.107863

[14] Remelgado, R. et al. A crop type dataset for consistent land cover classification in Central Asia. Sci Data 7, 250 (2020). https://doi.org/10.1038/s41597-020-00591-2

[15] Boryan, C., Yang, Z. W., Mueller, R. & Craig, M. Monitoring US agriculture: the US Department of Agriculture, National Agricultural Statistics Service, Cropland Data Layer Program. Geocarto Int 26, 341-358 (2011). https://doi.org/10.1080/10106049.2011.562309

[16] Fisette, T. et al. in 2013 Second International Conference on Agro-Geoinformatics 270-274 (IEEE, 2013).

[17] FAO. Crop Type (Afghanistan - 10m - 2020) - EOSTAT, FAO Map Catalog https://data.apps.fao.org/catalog/iso/3cf3376c-50fa-42a6-8bfe-070a56fd2112 (2021).

[18] Blickensdörfer, L. et al. Mapping of crop types and crop sequences with combined time series of Sentinel-1, Sentinel-2 and Landsat 8 data for Germany. Remote Sens Environ 269, 112831 (2022). https://doi.org/10.1016/j.rse.2021.112831

[19] Dong, J. et al. Early-season mapping of winter wheat in China based on Landsat and Sentinel images. Earth Syst Sci Data 12, 3081-3095 (2020). https://doi.org/10.5194/essd-12-3081-2020

[20] Qiu, B. W., Huang, Y. Z., Chen, C. C., Tang, Z. H. & Zou, F. L. Mapping spatiotemporal dynamics of maize in China from 2005 to 2017 through designing leaf moisture based indicator from Normalized Multi-band Drought Index. Comput Electron Agr 153, 82-93 (2018). https://doi.org/10.1016/j.compag.2018.07.039

[21] Qiu, B. W. et al. Maps of cropping patterns in China during 2015-2021. Sci Data 9, 479 (2022). https://doi.org/10.1038/s41597-022-01589-8

[22] Singha, M., Dong, J. W., Zhang, G. L. & Xiao, X. M. High resolution paddy rice maps in cloud-prone Bangladesh and Northeast India using Sentinel-1 data. Sci Data 6, 26 (2019). https://doi.org/10.1038/s41597-019-0036-3

[23] Zheng, Y., Luciano, A. C. D., Dong, J. & Yuan, W. P. High-resolution map of sugarcane cultivation in Brazil using a phenology-based method. Earth Syst Sci Data 14, 2065-2080 (2022). https://doi.org/10.5194/essd-14-2065-2022

[24] FAO. *Crop Type (Senegal - 10m - 2018) - EOSTAT, FAO Map Catalog* https://data.apps.fao.org/catalog/iso/5c377b2b-3c2e-4b70-afd7-0c80900b68bb (2021).

[25] Australian Bureau of Agricultural and Resource Economics and Sciences (ABARES). Land use of Australia 2010-11 to 2015-16, 250m, https://doi.org/10.25814/7ygw-4d64 (2022).

[26] Thierion, V., Vincent, A. & Valero, S. Theia OSO Land Cover Map 2021 (Version 1) Zenodo https://doi.org/10.5281/zenodo.6538910 (2022).

[27] Japan Aerospace Exploration Agency Earth Observation Research Center (JAXA EORC). *High Resolution Land-Use and Land-Cover Map of Japan (version 21.11)* https://www.eorc.jaxa.jp/ALOS/en/dataset/lulc/lulc_v2111_e.htm (2021).

[28] Li, H. *et al.* Development of a 10-m resolution maize and soybean map over China: Matching satellite-based crop classification with sample-based area estimation. *Remote Sens Environ* **294**, 113623 (2023).
